# Haplotype Diversity in mtDNA of Honeybee in the Czech Republic Confirms Complete Replacement of Autochthonous Population with the C Lineage

**DOI:** 10.3390/insects15070495

**Published:** 2024-07-02

**Authors:** Aleš Knoll, Lucie Langová, Antonín Přidal, Tomáš Urban

**Affiliations:** 1 Department of Animal Morphology, Physiology and Genetics, Faculty of AgriSciences, Mendel University in Brno, Zemědělská 1, 613 00 Brno, Czech Republic; 2 Department of Zoology, Fishery, Hydrobiology and Apidology, Faculty of AgriSciences, Mendel University in Brno, Zemědělská 1, 613 00 Brno, Czech Republic

**Keywords:** *Apis mellifera*, mitochondrial DNA, *tRNA^leu^-cox2*, *cox1*, introgression, population, A lineage

## Abstract

**Simple Summary:**

This research analyzed for the first time the diversity in the Czech population of the Western Honey Bee. It used two mitochondrial DNA markers to identify haplotypes. Sequencing revealed both common and novel haplotypes. The *cox1* sequences showed more diversity than the *tRNA^leu^-cox2* sequences. Most haplotypes belong to the C lineage. Some haplotypes showed links to the bees of African origin. No indigenous M lineage haplotype was found. Thus, the native population of the Dark Honey Bee, *Apis mellifera mellifera*, has been completely replaced by the C lineage. The haplotype networks and phylogenetic trees showed that some A and C lineage haplotypes were distinct. The haplotype diversity data indicate that there is ongoing population expansion and introgression. The Czech honeybee population is diverse, with different haplotypes in both *tRNA^leu^-cox2* and *cox1* sequences.

**Abstract:**

The study aimed to analyze the genetic diversity in the Czech population of *Apis mellifera* using mitochondrial DNA markers, *tRNA^leu^-cox2* intergenic region and *cox1* gene. A total of 308 samples of bees were collected from the entire Czech Republic (from colonies and flowers in 13 different regions). Following sequencing, several polymorphisms and haplotypes were identified. Analysis of *tRNA^leu^-cox2* sequences revealed three *Dra*I haplotypes (C, A1, and A4). The *tRNA^leu^-cox2* region yielded 10 C lineage haplotypes, one of which is a newly described variant. Three A lineage haplotypes were identified, two of which were novel. A similar analysis of *cox1* sequences yielded 16 distinct haplotypes (7 new) within the population. The most prevalent *tRNA^leu^-cox2* haplotype identified was C1a, followed by C2a, C2c, C2l, and C2d. For the *cox1* locus, the most frequent haplotypes were HpB02, HpB01, HpB03, and HpB04. The haplotype and nucleotide diversity indices were high in both loci, in *tRNA^leu^-cox2* with values of 0.682 and 0.00172, respectively, and in *cox1* 0.789 and 0.00203, respectively. The Tajima’s D values were negative and lower in *tRNA^leu^-cox2* than in *cox1*. The most frequent haplotypes were uniformly distributed across all regions of the Czech Republic. No haplotype of the indigenous M lineage was identified. High diversity and the occurrence of rare haplotypes indicate population expansion and continuous import of tribal material of the C lineage.

## 1. Introduction

The Western Honey Bee (*Apis mellifera Linnaeus*, 1758) is a species that originated either (a) in Africa and expanded into Eurasia at least twice [1] or through at least two pathways, across the Strait of Gibraltar and via Asia Minor [2] or (b) in Asia and the divergence between western and eastern European populations, which were likely derived from two distinct colonization routes [3] and similarly with other explanations and description of a new subspecies of M lineage from the eastern Tian Shan mountains [4] or with at least three expansions leading to African and European lineages [5]. As they expanded their range, climatic shifts and geographical barriers played a crucial role in shaping the sub-speciation patterns we observe today [6]. There is a core set of genes associated with worker and colony traits that facilitated the adaptive radiation of honeybees across their vast distribution [5].

The Western Honey Bee has been diversified into 31 subspecies [7], and up to seven evolutionary lineages have been identified [5] and characterized based on morphometric, molecular, ecological, ethological, and physiological traits [8,9]. The subspecies have a superior genetic diversity that enables them to adapt effectively to their environment. This adaptation includes withstanding various temperatures and altitudes [10]. Beekeepers’ practices, such as the intentional introduction of different honeybee subspecies and queens, have increased genetic diversity in *Apis mellifera* populations all over Europe [11]. European beekeepers have historically introduced various honeybee subspecies, leading to hybridization and the creation of new genetic clades [12]. Especially, the Carniolan Honey Bee (*Apis mellifera carnica* Pollman, 1879) is a subspecies of honeybee native to Europe, which has been introduced to various regions worldwide due to its favorable features for beekeeping [13].

The Czech Republic registers about 9 colonies per km^2^ [14], which is one of the highest colony densities all over the world [15,16]. There are registered about 8 beekeepers per 10 km^2^ (65,058 in total) and 162 inhabitants per beekeeper, which is the highest and the lowest values, respectively, in the world [16].

In most of the territory of today’s Czechia, the native lineage is M lineage (the Dark Honey Bee, *Apis mellifera mellifera* Linnaeus, 1758) [17,18] with the exception of the southernmost region of Moravia, where C lineage natively reached (the Carniolan race) [18,19,20,21]. Natural/accidental or intentional crossing of the native subspecies with other imported races on the Czech territory is well documented from the middle of the 19th century at the latest [22].

The mitochondrial genome of the western honeybee is approximately 16.5–17 kb [23]. The reference genome NC_001566 is 16,343 bp [24]. The complete mtDNA genome in the Carniolan race (MN250878) is 16,358 bp [25]. Mitochondrial DNA (mtDNA) has been extensively used as a molecular marker due to its maternal inheritance, rapid evolutionary rate, and lack of recombination. Analysis of a fragment of mitochondrial DNA located between the genes for cytochrome oxidase c subunits 1 and 2 (*cox1* and *cox2*) is the most used genetic method to distinguish subspecies and study bee variability [23,26,27]. By analyzing mtDNA sequences, researchers have revealed distinct lineages and haplotypes within the Carniolan subspecies [28,29].

The *tRNA^leu^-cox2* intergenic region sequence consists of two mitochondrial genes: transfer RNA (tRNA)-Leu and cytochrome oxidase subunit II (*cox2*). This fragment contains a high level of AT and shows significant differences in nucleotide length and composition between honeybee populations [23]. This amplicon is cleaved by the *Dra*I restriction endonuclease, which specifically recognizes the TTTAAA sequence, to determine the lineage [6]. The intergenic region can be used to distinguish between the three basic bee lineages, A, M, and C. This non-coding region contains the variable-length P and Q fragments [23]. Two variants of the P_0_ and P fragments, which differ by a short deletion, distinguish the A and M lineages. Both can then have up to four tandem repeats of the Q sequence. The C lineage has only one Q sequence [6,30].

Numerous studies have investigated the genetic diversity of the mtDNA *tRNA^leu^-cox2* region in *A. mellifera* populations, revealing informative insights about the evolutionary history of honeybees. A study published in 2023 [31] analyzed mitochondrial DNA (mtDNA) haplotypes and their distribution in *Apis mellifera* populations in the United States. The *tRNA^leu^-cox2* mtDNA region of the honeybee has also been investigated in other countries, where *A. m. carnica* bees are reared. [30,32,33,34,35].

DNA barcoding is a commonly used method of species identification based on the mitochondrial DNA sequence of the *cox1* gene. This is compared to sequences stored in databases like the BoldSystem and GenBank [36,37]. This DNA fragment is highly conserved within a taxon and is often used to distinguish between taxa. Its variability can also be used to assess diversity. It is important to note that despite ongoing efforts, the ability to distinguish subspecies based on this fragment is limited [38].

The main goal of this study was to examine the genetic diversity of the Czech population of western honeybees by analyzing two mitochondrial DNA markers: the *tRNA^leu^-cox2* intergenic region and the *cox1* gene region commonly used for insect barcoding. By identifying haplotypes and assessing intra-population diversity, the study aimed to determine the specific haplotypes of the western honeybee in the Czech Republic. Additionally, the research sought to investigate potential regional differences, the importation of other races from abroad, and the presence of autochthonous remnants of the Dark honeybee in the western parts of Bohemia.

## 2. Materials and Methods

### 2.1. Sampling of Bees

The Czech Republic is divided into 76 districts, which are distributed relatively evenly across the country. The average size of one district is 1024 km^2^. Districts are divided among 13 regions. The Capital City of Prague is not included in any particular district. In order to sample bees evenly, we took randomly in each district, including the Capital City of Prague: (a) two samples from hives (beekeeper and the origin of bees are known) and (b) two samples from flowers (beekeeper and the origin of bees are unknown). A total of 308 samples were obtained during 2022–2023. The hive sample consisted of five worker bees, which were taken from brood comb shortly before emerging to be sure that the sampled bees were daughters of the same queen. Each of the two hive samples was taken from different beekeepers in different parts of the same district. The flower samples were obtained by catching 10 free-flying bees on flowers. Each of the two flower samples was obtained in two distant locations within the same district, with the samples being collected at a sufficient distance from an apiary to ensure minimal duplication.

After the sampling, the material was stored at a temperature of −20 °C until the DNA isolation.

### 2.2. DNA Extraction

Genomic DNA was extracted using a standard protocol (Tissue Genomic DNA Mini Kit, Geneaid Biotech Ltd., New Taipei City, Taiwan). Genomic DNA was extracted from thoracic muscle tissues.

### 2.3. PCR Amplification

For mtDNA sequencing analysis, we always selected two random samples from different apiaries from each district and similarly two from flowers within our bee genetic bank. PCR was carried out in an amount of 1 µL of isolated DNA. Fragment of mtDNA *tRNA^leu^-cox2* intergenic region was amplified using specific primers E2: 5′ GGCAGAATAAGTGCATTG 3′ and H2: 5′ CAATATCATTGATGACC 3′ [39]. The sequenced region encompasses the 3′ end of the *cox1* gene, the *tRNA^Leu^* gene, an intergenic region of variable length, and the 5′ end of the *cox2* gene. PCR was performed using a thermocycler Biometra Trio 48 in a total volume of 10 μL containing 1X Combi PPP Master Mix (Top-Bio, Vestec, Czech Republic), which contained 0.5 U of hot star Taq polymerase, 200 μM total dNTP and 2.5 mM MgCl2, and 0.1 μM each primer and 2 μL of DNA isolate. The cycling conditions were as follows: 95 °C (2 min); 33 cycles of a 45 s denaturation at 95 °C, a 40 s annealing at 46 °C, a 40 s elongation at 62 °C; and a final extension step at 62 °C for 15 min.

We also used for analysis the internal fragment of the *cox1* gene used for barcoding, amplified with universal primers according to [40] with modifications based on the honeybee genome sequence (NC_061380): ApLCO: 5′ TATCAACCAATCATAAAAATATTGG 3′ and ApHCO: 5′ TAAACTTCTGGATGACCAAAAAATCA 3′. The PCR was carried out using the same thermocycler and the same reaction mix as described above and under the following cycling conditions: 95 °C (2 min); 32 cycles of a 30 s denaturation at 95 °C, a 30 s annealing at 50 °C, a 40 s elongation at 62 °C; and a final extension step at 72 °C for 20 min.

### 2.4. DNA Sequencing

The obtained PCR products were verified using agarose gel electrophoresis and sequenced according to the standard Sanger sequencing methodology on the genetic analyzer ABI PRISM 3500. Each sample was sequenced on both sides using E2 and H2 primers for *tRNA^leu^-cox2* and ApLCO and ApHCO primers for *cox1* sequences. The obtained sequences were analyzed and compared using the SeqScape software v4.0. The *tRNA^leu^-cox2* and *cox1* sequences are available in FASTA format in File S1 and File S3, respectively.

### 2.5. Data Analysis

Evolutionary lineages were determined from the *tRNA^leu^-cox2* intergenic region structure described by [23,41]. The *Dra*I restriction spectra of the *tRNA^leu^-cox2* fragment sequences were performed in silico using the Unipro UGENE ver. 49.1 [42]. The *cox1* sequence, which is widely used in the Barcoding organism identification method, was verified using the BOLD database (https://boldsystems.org/index.php/IDS_IdentificationRequest, accessed on 14 March 2024).

For detection of DNA polymorphisms and haplotypes in both mtDNA regions using all sequences from a multiple sequence alignment, DnaSP ver. 6.12.03 was used [43]. The nucleotide substitutions and insertions/deletions for each haplotype were determined by comparison of positions with our primer-free sequences and the NC_001566 genome reference [24].

The haplotypes identified in the two regions were characterized by means of multiple sequence alignment using the Kalign [44]. For the *tRNA^leu^-cox2* region, the alignment had to be corrected manually due to the presence of the P_0_ sequence and the duplication of the Q sequence in three haplotypes. Multiple sequence alignments in Clustal format can be found in File S2 and File S4.

To identify specific *tRNA^leu^-cox2* haplotypes of C and A lineages, reference sequences with 100% identity were searched (using BLAST, https://blast.ncibi.nlm.nih.gov/Blast.cgi, accessed from 5 January 2024 to 15 April 2024) in the National Center for Biotechnology Information (NCBI) nucleotide database (GenBank). BLAST was used to verify the *cox1* haplotypes. The *cox1* sequence was also verified using the BOLD database (https://boldsystems.org/index.php/IDS_IdentificationRequest, accessed from 15 March 2024 to 25 April 2024). The new and previously published *tRNA^leu^-cox2* and *cox1* haplotype sequences have been deposited in GenBank.

Genetic diversity indices, including nucleotide diversity π [45], haplotype diversity [46], and Tajima’s D for testing the neutral mutation hypothesis [47], were assessed using the pegas package (v. 1.3) [48] in the R program (v. 4.3.2, R Foundation for Statistical Computing, Vienna, Austria) [49].

Phylogeographic haplotype networks were constructed using 308 mtDNA haplotypes for both loci, *tRNA^leu^-cox2* and *cox1*, in 14 geographical regions in the Czech Republic. The phylogeographic networks were generated using the randomized minimum spanning tree method with 1000 iterations by rmst function in the pegas package (v. 1.3 [48]) of R v. 4.3.2.

The construction of phylogenetic trees to determine the evolutionary relationships among *A. melifera* haplotypes (*tRNA^leu^-cox2* and *cox1*) was inferred by using the maximum likelihood method and Tamura–Nei model [50] in program Mega X [51]. The bootstrap consensus tree was derived from 10,000 replicates. Branches corresponding to partitions reproduced in less than 50% of bootstrap replicates were collapsed. The percentage of replicate trees in which the associated haplotypes clustered together in the bootstrap test (10,000 replicates) is shown next to the branches [52]. Initial trees for the heuristic search were obtained automatically by applying Neighbor-Join and BioNJ algorithms to a matrix of pairwise distances estimated using the Tamura–Nei model and then selecting the topology with the highest-level log likelihood value. The final data sets for the *tRNA^leu^-cox2* and *cox1* regions were 13 and 16 nucleotide sequences, respectively, with a total of 796 and 658 positions.

## 3. Results

### 3.1. Analysis of tRNA^leu^-cox2 Sequences

**Figure 1:**
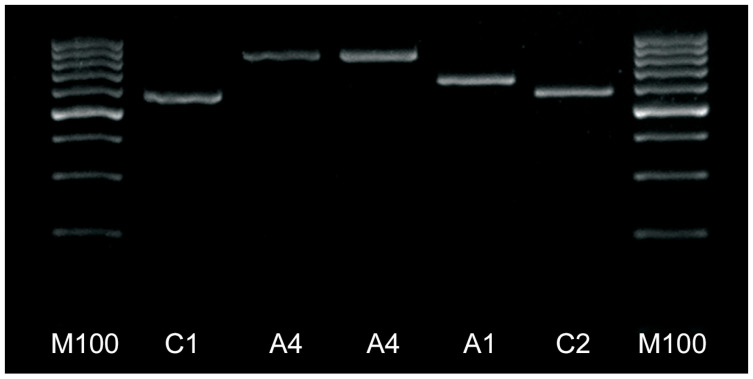
PCR product of the COI-COII region of mitochondrial DNA by agarose gel electrophoresis. M: 100 bp DNA ladder, C1 and C2 (Q), A4 (P_0_QQ), and A1 (P_0_Q) lineages.

The result of PCR amplification of the *tRNA^leu^-cox2* sequence is shown in Figure 1. PCR amplicons show visible differences in size due to the internal structure of each haplotype determined by the presence of P_0_ and Q sequences. The differences in sequence length sizes are due to indels and variability in the intergenic region by insertion of the P_0_ and a tandem repeat of the Q sequences identified after the *Dra*I test and sequencing. Detailed positions are provided in File S2.

The *Dra*I test (in silico) identified two evolutionary lineages, C and A, in the Czech Republic samples. Three out of 308 samples were found to belong to lineage A. One sample had the P_0_Q variant, and two samples had the P_0_QQ variant (Table 1).

After sequencing, all haplotypes were validated by BLAST, and homology to known sequence variants was verified. We identified the haplotypic variants based on 100% similarity. Three samples (C2d7, A1ha, and A4na) did not match in the database and are therefore considered to be novel (Table 1).

### 3.2. Analysis of Polymorphisms of tRNA^leu^-cox2

A comparison of all obtained *tRNA^leu^-cox2* sequences revealed 21 polymorphic sites, including 13 SNPs and 8 indels. There were four singleton variable sites at positions 25, 254, 272, and 274. Additionally, there were five variable indel sites (site positions: 115, 161, 183, 256, and 290). In the gaps, four variable sites were found (only in A lineage in P_0_ and in the second Q fragment; site positions: 81, 308, 347, and 372). The parsimony informative sites were 8 and they were located at positions 112, 201, 202, 511, 586, 640, 646, and 695. The sequences of all found haplotypes were deposited in Genbank (accession numbers in Table 1) and are also clearly presented in File S1. Multiple alignment of haplotypes and localization of polymorphic sites are included in File S2.

Analysis of the sequence data in the intergenic region identified 10 different haplotypes belonging to the C lineage (with a single Q sequence). Sequence alignment of the C-lineage haplotypes revealed five SNPs and five indels. Furthermore, three haplotypes belonging to the A lineage were found (A1ha—P_0_Q; A4s and A4na—P_0_QQ).

### 3.3. Description of New Haplotypes

New haplotypes have been found that have not been published before. The newly designated haplotype C2d7 was most similar (99.81%) to the reference sequence JF723977.1 referred as C2d. A deletion of the T nucleotide corresponding to position 25 in the reference sequence was found in our sequence. Our sequence contains a deletion at position 3412 bp within the gene *tRNA^leu^* compared to the genome-wide sequence (MN250878.1 [25]). For verification, the amplification and sequencing of this sample was repeated.

The haplotype newly designated A1ha was similar (99.83%) to the reference KX463739.1 (A1h). The only difference was the C/T substitution at position 230 in the reference. The novel haplotype A4na was similar (99.75%) to KX463793.1 (A4n). Two substitutions at positions 313 and 347, A/C and C/T, respectively, were different.

### 3.4. Analysis of cox1 Sequences

The *cox1* fragment was sequenced in 308 samples of 658 bp each. Twenty-two polymorphic sites were identified, of which 10 were singleton variable sites (site positions according to File S4: 52, 259, 271, 274, 285, 389, 421, 437, 442, and 571) and 12 parsimony informative sites (site positions: 1, 99, 100, 133, 142, 196, 235, 310, 448, 548, 641, and 643).

A total of 16 haplotypes (HpB01–HPB16) were identified and their sequences were deposited in the GenBank repository with accession numbers PP401657–PP401672. These sequences are also listed in File S3. A multiple alignment with site positions can be found in File S4.

Haplotypes HpB01–HpB04 are the most common in the Czech Republic, and at the same time, 100% identity was found with sequences in GenBank belonging to many subspecies belonging to lineage C (e.g., *A. m. carnica*, *A. m. anatoliaca*, *A. m. ligustica*). Haplotypes HpB06–HpB12 did not have 100% identity with sequences in GenBank, so they are newly discovered and are among the less common. All the samples were identified as *Apis mellifera* by the BOLD system. In our three samples (HpB14–HpB16), which were assigned to lineage A based on *tRNA^leu^-cox2* haplotypes, we found similarities with bees of African origin in the NCBI and BOLD databases for *cox1* haplotypes. The *cox1* haplotypes HpB14 and HpB15 had 100% and HpB16 had 99.85% similarity to the NCBI references of African origin (MT871137.1, KF833379.1, and MN714162.1, respectively). The HpB16 haplotype is newly described.

Supplementary Table S2 displays the most common haplotype combinations of *tRNA^leu^-cox2* and *cox1* sequences. The majority of combinations were C1a and HpB02, C1a and HpB03, C2c and HpB03, C2e and HpB01, C2l and HpB04, and C2d and HpB02. Other haplotype combinations were less frequent. Rare haplotypes of the African lineage had combinations of A1ha and HpB14, A4na and HpB16, and A4s and HpB15, but only in one sample each. The individuals with *tRNA^leu^-cox2* haplotypes C2e, C1a, C2c, C2d, and C2l exhibited the most divergent *cox1* haplotypes. A highly significant statistical difference (X-squared = 1715.4, df = 180, *p*-value < 2.2 × 10^−16^) was found between the frequency combinations of the two haplotype sequences.

### 3.5. Distribution of Haplotypes Based on the Sampling Technique

Bee samples were gathered from hives and flowers. Four *tRNA^leu^-cox2* haplotypes (C2j, C2s, A1ha, and A4s) were exclusively found in hive samples, while four other haplotypes (C2d7, C2i, C2y, and A4na) were only found in flower catch samples. Both types of samples had a similar number of specific *tRNA^leu^-cox2* haplotypes (Table 2). Haplotypes C1a, C2l, C2e, and C2c were present in both hive and flower catch samples at similar frequencies. The only difference observed between the two types of collections was the presence of rare haplotypes, which was not statistically significant (X-squared = 16.562, df = 12, *p*-value = 0.1668).

The distribution of haplotypes in the *cox1* sequence shows that among the 16 haplotypes, HpB01–HpB04 were the most abundant and had similar frequencies among the groups based on the type of collection (Table 3). The remaining haplotypes were present in small numbers, some of which were found only in hives (HpB11–HpB15) or caught on flowers (HpB06, HpB08, HpB09, HpB10, and HpB16). There were no statistically significant differences between hive or flower sampling (X-squared = 21.705, df = 15, *p*-value = 0.1158). Therefore, we will not consider the type of sampling in subsequent analyses, such as haplotype networks and phylogenetic trees.

### 3.6. Haplotype Diversity

The values for haplotype and nucleotide diversity as well as Tajima’s D are shown in Table 4. The haplotype diversity parameters (Hd and π) and Tajima’s D were higher in the hive samples compared to the catch-on-flower samples for both *tRNA^leu^-cox2* and *cox1* haplotypes. A higher value of π indicates that the haplotypes detected in the hives are more divergent than those in the catch of flowers.

Comparison of haplotype diversity and Tajima’s D parameters between *tRNA^leu^-cox2* and *cox1* haplotypes reveals higher diversity in *cox1* haplotype sequences than in *tRNA^leu^-cox2* haplotype sequences. Negative Tajimas’s D values in both sequences indicate that the honeybee population in the Czech Republic has undergone or is undergoing population expansion, with the emergence of less abundant and new haplotypes.

### 3.7. Haplotype Networks for tRNA^leu^-cox2 and cox1 Haplotypes and Regions in the Czech Republic

**Figure 2:**
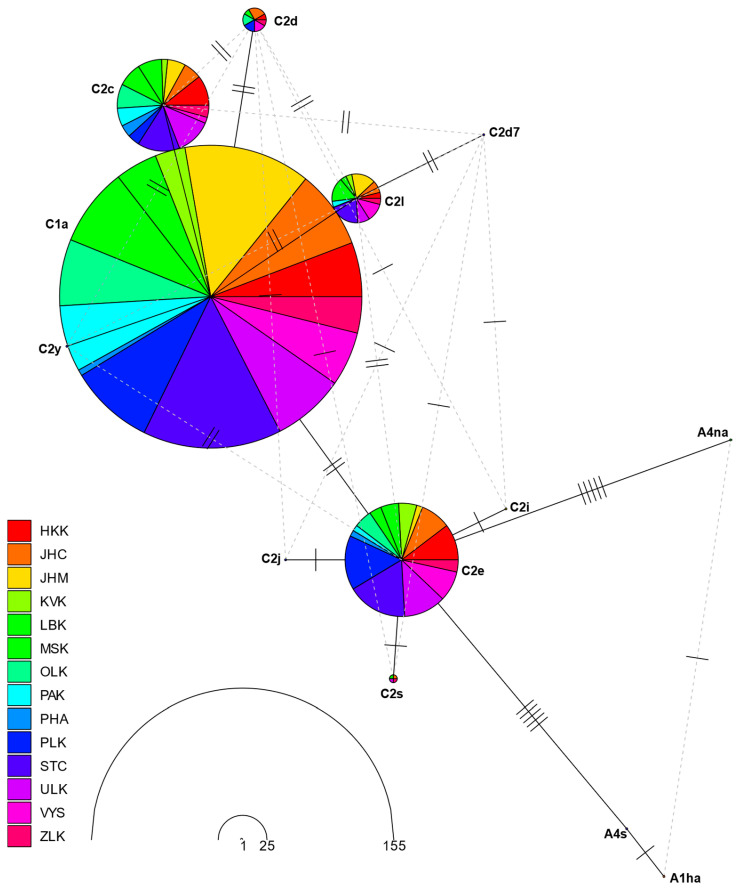
RMST haplotype network analysis among studied 308 *tRNA^leu^-cox2* sequences. The size of the circles is proportional to the number of individuals and the different colors represent different regions (HKK—Hradec Králové Region, JHC—South Bohemia Region, JHM—South Moravia Region, KVK—Karlovy Vary Region, LBK—Liberec Region, MSK—Moravian-Silesian Region, OLK—Olomouc Region, PAK—Pardubice Region, PHA—Capital City of Prague, PLK—Plzeň Region, STC—Central Bohemia Region, ULK—Ústí Region, VYS—Vysočina Region, ZLK—Zlín Region).

**Figure 3:**
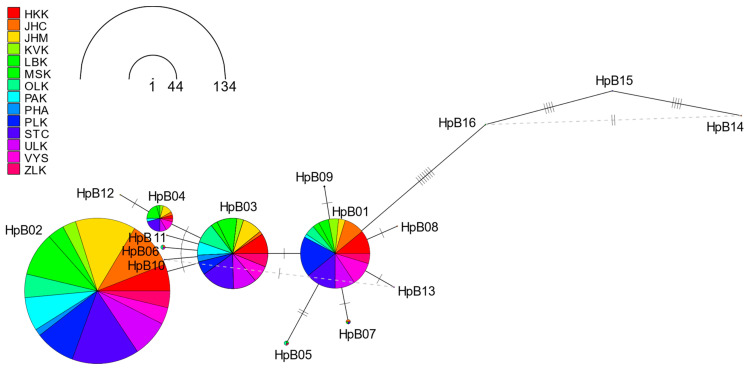
RMST haplotype network analysis among studied 308 *cox1* sequences. The size of the circles is proportional to the number of individuals and the different colors represent different regions (HKK—Hradec Králové Region, JHC—South Bohemia Region, JHM—South Moravia Region, KVK—Karlovy Vary Region, LBK—Liberec Region, MSK—Moravian-Silesian Region, OLK—Olomouc Region, PAK—Pardubice Region, PHA—Capital City of Prague, PLK—Plzeň Region, STC—Central Bohemia Region, ULK—Ústí Region, VYS—Vysočina Region, ZLK—Zlín Region).

RMST (the randomized minimum spanning tree) haplotype networks illustrating the frequencies and relationships of the *tRNA^leu^-cox2* and *cox1* haplotypes found in the Czech Republic are shown in Figure 2 and Figure 3, respectively.

The RMST haplotype network, based on *tRNA^leu^-cox2* sequences from 308 samples, revealed that the haplotypes of the C lineage were grouped together, while those of the A lineage were distinctly separated from them. The central part contains two main haplotypes, C1a and C2e, to which the other haplotypes are linked. C1a is linked to C2c, C2d, C2y, C2l, and C2d7, while C2e is linked to C2j, C2i, and C2s, and is five mutations away from the A lineage haplotypes (A4s, A1ha, and A4na).

The haplotypes that occurred most frequently were C1a (50.3%), C2e (18.8%), C2c (15.3%), and C2l (8.1%). These haplotypes were evenly distributed in all regions. The remaining haplotypes, due to their low frequency, were unevenly distributed across regions. Notably, eight haplotypes, including all A lineage haplotypes, were observed only once in different regions (Table S1a).

A haplotype network was constructed by analyzing *cox1* haplotypes from 308 samples (Figure 3). The central position is occupied by haplotype HpB01, to which HpB05, HpB05, HpB08, HpB09, HpB13, and HpB03 are linked. Haplotype HpB03 forms a second center, to which haplotypes HpB02, HpB04, HpB06, HpB10, and HpB11. HpB04 is linked to the HpB12 haplotype. Haplotypes HpB14, HpB15, and HpB16 identified in samples belonging to A lineage by *tRNA^leu^-cox2* sequence were also linked to HpB01.

The *cox1* sequence had four predominant haplotypes, namely, HpB02 (43.5%), HpB03 (21.1%), HpB01 (20.8%), and HpB04 (7.18%), and their occurrence was uniform across all regions. The less common haplotypes, HpB05–HpB016, were found in various regions, including JHC, STC, ULK, MSK, OLK, LBK, and ZLK (Table S1b).

### 3.8. Phylogenetic Analysis

**Figure 4:**
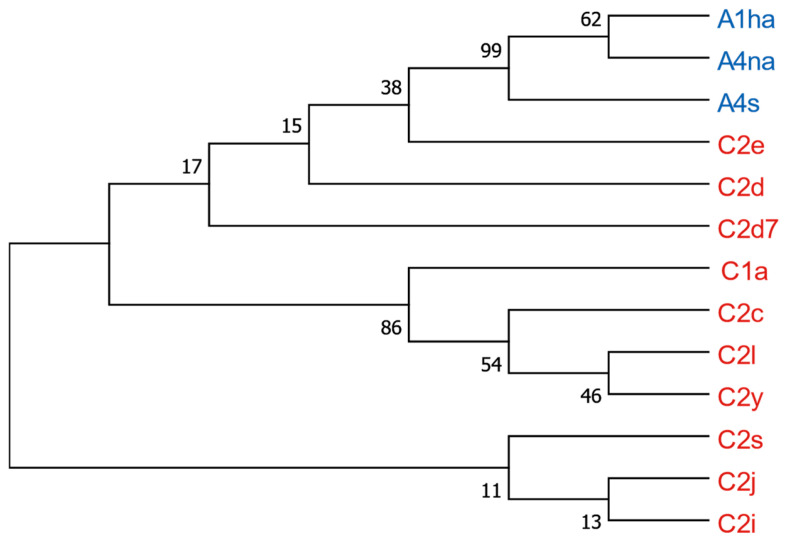
Bootstrap consensus tree of *tRNA^leu^-cox2* haplotypes in the Czech Republic by using the maximum likelihood method and Tamura–Nei model. Besides the branches is the percentage of replicates where the corresponding haplotypes were found to cluster (the bootstrap test—10,000 replicates). The analysis included 13 nucleotide sequences. A total of 796 positions were included in the final dataset. C lineage haplotypes are in red and A lineage haplotypes are in blue.

The identified haplotype sequences of *tRNA^leu^-cox2* and *cox1* were aligned, and maximum likelihood phylogenetic trees were constructed. This method also allowed for the differentiation of haplotypes of *tRNA^leu^-cox2* between the A and C lineages (Figure 4). The new haplotype C2d7 is clustered with the haplotypes C2d and C2e, which are the nearest to the haplotypes of the A lineage (A4s, A1ha, A4na). The next cluster included haplotypes C1a, C2c, C2l, and C2y. The last cluster consisted of haplotypes C2s, C2j, and C2i.

**Figure 5:**
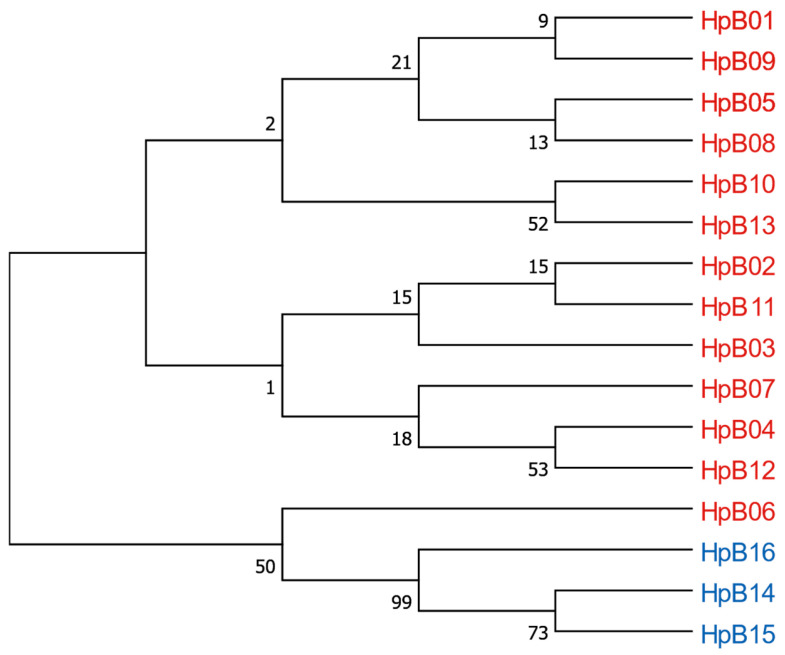
The bootstrap consensus tree *cox1* haplotypes in the Czech Republic by using the maximum likelihood method and Tamura–Nei model. Besides the branches is the percentage of replicates where the corresponding haplotypes were found to cluster (the bootstrap test—10,000 replicates). The analysis included 16 nucleotide sequences. A total of 658 positions were included in the final dataset. The color coding of the *cox1* haplotypes corresponds to the lineages identified in Figure 4 based on the *tRNA^leu^-cox2* haplotypes (red for the C lineage and blue for the A lineage).

The coding sequence of *cox1* shows an evolutionary pattern that is similar to the one described for the *tRNA^leu^-cox2* sequences (see Figure 5). The bootstrap consensus tree reveals three clusters. The first cluster includes haplotypes HpB01, HpB09, HpB05, HpB08, HpB10, and HpB13. The second cluster contains the haplotypes HpB02, HpB11, HpB03, HpB07, HpB04, and HpB12, as well as HpB06 (which differs from HpB03 by only one substitution). The third cluster contains three haplotypes, HpB14, HpB15, and HpB16, which are found in samples belonging to the A lineage according to the *tRNA^leu^-cox2* sequence.

The phylogenetic tree analyses produced results that are comparable to those of the haplotype networks.

## 4. Discussion

In the representative dataset evenly covering the entire territory of the Czech Republic, no specimens of the indigenous Dark bee were found. This is consistent with trends in the Czech territory in the previous century described by Tomšík [19] and later announced by Veselý [21] both with morphometry. We confirmed on the molecular data from mtDNA that the indigenous honeybee population of the M lineage was replaced by a population belonging to the C lineage. This is probably the result of the importation of honeybees from the more southern parts of Europe, which historically began already in the middle of the 18th century [18,53] and then continued intensively in the second half of the 19th century [22] in the Czechia territory. We reflect on suggestions resulting from the screening of several apiaries near to borderline with Germany and Austria in Šumava and Novohradské mountains in 2010 [54,55]. There were found traces of the Dark bee with the morphometry. Exterior of some workers and drones were conspicuously similar to the Dark honeybee. Finding the mitochondrial haplotype of the Dark honeybee nowadays has a low probability when the Czech bee population has been replaced by the Carniolan bee with grading-up [21]. Traces of the Dark bee were repeatedly found with morphometry even in the 1960s and 1970s [21,56], and the Bohemian population was morphometrically closer to the Dark honeybee and the Moravian one to the Carniolan honeybee [57]. We suppose that more detailed screening of the nuclear molecular data of the honeybee populations near the southwest border line of the Czechia could reveal traces of the Dark bee.

In this study, *tRNA^leu^-cox2* haplotypes were identified and their frequencies were determined for the first time in the Czech Republic. Given the probable origin of the bees, it is possible to make a comparison with the following countries where C lineage occurs.

In the Serbian population (*A. m. carnica*), the most prevalent haplotype is C2d [58], which, in Czechia, was the fourth most common haplotype, after haplotypes C1a, C2e, and C2c. The second most common haplotype is C2e, the same as in our country, and the third is C1a, which was the most common in Czechia. Haplotype C2j is described as rare, as the same was in Czechia. The study conducted by Muñoz et al. [33] revealed that C2e was the most prevalent haplotype within the *A. m. carnica* population in Croatia. The frequency of C2e was 0.45, while the frequencies of C1 and C2c were 0.35 and 0.15, respectively. These three haplotypes also represent the most abundant haplotypes in the Czech Republic. However, the number of samples analyzed for the Croatian study was somewhat limited (N = 20).

A study [59] on honeybees from central and southeastern Europe, including Italy, Austria, Slovenia, Hungary, Croatia, Bosnia, Montenegro, Albania, Serbia, Romania, and Greece, found that the frequency of haplotypes varied across the region. The most prevalent haplotypes were C2d (0.39), C2c (0.24), and C1a (0.11). In the Czech Republic, the most abundant haplotype was C1a, which should be typical of *A. m. ligustica* populations.

In Russia, the C2c haplotype and C2j haplotype have been identified most frequently within the C lineage [35]. Haplotype C2c represents the third most frequent haplotype in the Czech Republic, while haplotype C2j was observed in the Czech Republic, as above already mentioned, only on a single occasion; therefore, it can be concluded that its occurrence is very sporadic. Furthermore, their research indicated a notable influx of C lineage into the original populations of M lineage.

In the USA, [31] identified the most prevalent haplotype within the C lineage populations as C1 (corresponding to C1a), as observed in our study, and C2j, which was uncommon in the Czechia as above mentioned. Furthermore, Kaskinova et al. [35] indicated that haplotype C2j is characteristic of *A. m. caucasia*. In contrast, Alburaki et al. [31] identified the same haplotype characterizing the *A. m. carnica* subspecies. These different results suggest that the assignment of a specific *tRNA^leu^-cox2* haplotype to some C or O honeybee lineages is not possible to do with certainty.

Three found haplotypes belonged to A lineage; thus, we confirmed the introgression of African genes also in the Czech honeybee populations. A similar situation was described in Poland and Hungary [30]. These authors examined the influence of maternal origin in Central-East Europe, encompassing northern Poland, Hungary, and Romania. The highest frequency was found for the C lineage (0.881), followed by the M lineage (0.103) and A lineage (0.016). The proportion of bees of African origin was determined to be 1.64%, which is consistent with the findings of the present study because the frequency of A lineage in Czechia was approximately 1%. Therefore, it can be concluded that bees of African origin are gradually being introduced by beekeepers, and the trends described by Oleksa et al. [30] are also occurring in the Czech Republic.

In accordance with the authors’ arguments [30], the occurrence of African genes in Czechia cannot be considered as a result of the natural gene flow as it is recorded, e.g., in the Iberian Peninsula [26]. We also found haplotypes that were not recorded in the Iberian Peninsula [26] and are typical of the honeybee population in Sub-Saharan Africa [60]. We would like to note, besides Oleksa’s narrative about the possible origin of the African haplotypes from the Buckfast strain detected in their samples, that they did not mention another possible source: strain Elgon [61]. Elgon originates partially from the Sub-Saharan race *Apis mellifera monticola*, taken from Kenya to Sweden in 1989. The strain results from crossing *A. m. monticola* with the Buckfast strain with *A. m. sahariensis* (from Morocco). The Elgon strain is used also by several beekeepers in Czechia. One sample from our dataset was declared by a beekeeper as the Buckfast strain (detected as A1ha haplotype) and the second one as the Elgon strain (detected as A4s), indicating that the importation of strains has had an effect. The third sample of the A lineage (A4na) was caught on flowers in a different district, thus precluding the determination of the breeding origin. Given the history of breeding for over a century, the current official strategy of using only *A. m. carnica*, and the breeding saturation in the Czech Republic (the extremely high territorial density of bee colonies [14]), it is also likely to be a breeding import rather than a natural occurrence. Additionally, haplotypes A1ha and A4na are novel haplotypes that have not yet been published in the NCBI GenBank database. The sequences with the highest percent similarity were as follows: for A1ha, KX463739.1 (99.83%) and for A4na, KX463793.1 (99.75%).

A study on the haplotype composition of *A. m. carnica* in Slovenia and to a lesser extent in other countries was conducted [32], including the Czech Republic (N = 9). Their findings revealed the presence of two highly prevalent haplotypes, C1 and C2c, which are also among the most common haplotypes observed in the present study.

Distinctly high dominance of haplotype C1a (0.948, N = 58) was found in Italy [58], and Muñoz and De la Rúa [59] stated that this haplotype probably got into the Carniolan bee population through introgression from the population of the Italian honeybee common throughout northern Italy. A similar situation takes place in Hungary [34] where the Carniolan honeybee also occurs. The origin of the C1a haplotype in the Czech population can come from southern Carniolan populations imported into Czechia in the previous and last century [21,53,62,63] or from direct import of the Italian honeybee as it was reported already by Cori [22].

A novel haplotype (C2d7) was identified in a single sample belonging to the C lineage. A deletion was observed in the TTTTT sequence within the 3408–3412 bp region of the genome-wide sequence (MN250878.1, [25]), which is located within the *tRNA^leu^* gene. These positions correspond to positions 25–29 in the multiple sequence alignment (Kaling). This mutation has not been previously described in the C lineage (not found in NCBI GenBank). Nevertheless, the same mutation has been identified in certain haplotypes belonging to A (KC149980.1, MT176018.1, MT176002.1, and FJ477983.1) and O lineages (FJ743633.1).

For the first time, the Folmer region of the mitochondrial gene *cox1*, which has been used for species determination (by BOLD), was employed to study the variability of bees in Czechia. The results indicated high variability in this marker, with the identification of 16 haplotype variants.

Genetic diversity of the *cox1* fragment was also studied in Hungary [34], where they identified seven haplotypes of the C lineage in *A. m. carnica*. However, these sequences are shorter (345 bp) than our sequences (658 bp) and cover the internal part of our fragment. A comparison of this portion of the *cox1* gene revealed that the most prevalent haplotype, H9, is identical to our haplotypes HpB01 and HpB03, which collectively account for 41.9% of our samples. The second most prevalent haplotype, H2, is identical to HpB04, representing 7.8% of our samples. The third most prevalent haplotype, H10, is identical to our HpB02, which is present in 43.5% of the samples from Hungary.

The *cox1* haplotypes identified in this study (HpB01, HpB03, HpB02, and HpB13) match those previously identified by [64] (H1, H2, H2-1, and H1-1, respectively). The haplotype H1 was identified in the subspecies *A. m. caucasia*, H2 with *A. m. carpatica* (a Carpathian ecotype of the Carniolan race [18]), H2-1 with *A. m. carpatica*, and H1-1 with *A. m. caucasia*. However, all their haplotypes were also detected in our samples from the Czech honeybee population, which is a population of *A. m. carnica.* Therefore, we cannot confirm the race specificity of selected *cox1* haplotypes to any race.

In a study in Russia [65], authors proposed to distinguish subspecies according to the *cox1* sequence at positions 99 and 448 and developed the RFLP test. Our samples, belonging to *A. m. carnica*, had both A and G at position 99 (File S4); therefore, we could not confirm the possibility of using this detection procedure for the Czech population. On the other hand, our data show that it is not easy to assign mtDNA haplotypes to honeybee subspecies.

The haplotype and nucleotide diversity in honeybees identified as *A. m. carnica* were found for loci *tRNA^leu^-cox2* to be significantly lower (0.296 and 0.0009, respectively) in Hungary [34] than in our samples. In contrast, similarly high haplotype diversity (0.756 and 0.67) has been observed in the same subspecies by [59], as well as by [66], respectively.

High values (0.719 and 0.0020) were found in our population when analyzing haplotype and nucleotide diversity within the *cox1* sequence. On the contrary, lower values of haplotype diversity (0.551 and 0.371) and nucleotide diversity (0.001 and 0.0005) were found for the two C lineage mitotypes in Russia [64].

Results showed that the level of genetic variability based on the sequence of *tRNA^leu^-cox2* and *cox1* within and among districts is high unlike the Carniolan honeybee population in Slovenia [32]. The Slovenian honeybee population can be considered as an indigenous gene pool within the Carniolan population. The Czech one is less homogeneous with respect to the high value of haplotype and nucleotide diversity and negative values of Tajima’s D, and that is why we assume introgression from other honeybee races.

## 5. Conclusions

This study represents the first comprehensive examination of the diversity of the honeybee population in the Czech Republic. Sequencing revealed both common and novel haplotypes in two loci in mtDNA. Several haplotypes with high frequencies at both loci were identified (5 out of 13 haplotypes for *tRNA^leu^-cox2*; 4 out of 16 haplotypes for *cox1*), but also a considerable number of haplotypes with minimal frequencies, indicating a continual import of C lineages into the Czech Republic. No sequence belonging to the Dark bee, *A. m. meliffera*, was found. This evidence indicates that the autochthonous Dark bee population has been replaced by *A. m. carnica* over a period of more than 200 years due to grading-up to the Carniolan race. Additionally, three haplotypes of the A lineage were introduced by beekeepers with hybrid queens derived from Buckfast and/or Elgon strains. The results of the study indicated that the Czech population exhibited a high degree of genetic diversity in two mitochondrial DNA regions. The haplotypes with the highest frequencies were distributed relatively evenly across all regions. There is likely no significant genetic differentiation between regions within the country. This may be attributed to the type of beekeeping and breeding and the high prevalence of smallholder beekeeping in the Czech Republic. Our data can be utilized for future comparisons with populations in other countries. These findings offer valuable insights into genetic diversity and lineage and haplotype composition of honeybee populations in the Czech Republic for future comparative studies and trend analysis. For a more detailed description of diversity, analysis of microsatellite loci in nuclear DNA is desired.

## Figures and Tables

**Table 1:** *tRNA^leu^-cox2* mitochondrial DNA segment, evolutionary lineage (L), genetic structure (G).

L	G	Haplotype	NCBI Accession	*Dra*I Spectrum (bp)	Seq. Length (bp)	NCBI Reference	
						Accession Number (% Similarity)	Designated as
C	Q	C1a	PP350804	47, 41, 64, 420	572	JQ977699.1 (100)	*carnica*
C	Q	C2l	PP350810	47, 40, 64, 419	570	OR761864.1 (100)	*carnica*
C	Q	C2e	PP350812	47, 40, 63, 420	570	JQ977702.1 (100)	*carnica*
C	Q	C2d	PP350805	47, 40, 64, 420	571	JF723977.1 (100)	*carnica*
C	Q	C2c	PP350806	47, 40, 64, 420	571	JF723976.1 (100)	*carnica*
C	Q	C2j	PP350807	47, 40, 64, 420	571	KX463941.1 (100)	*carnica*
C	Q	C2s	PP350808	47, 40, 63, 421	571	JF723979.1 (100)	*carnica*
C	Q	C2d7 *	PP397042	46, 40, 64, 420	570	JF723977.1 (99.81)	*carnica*
C	Q	C2i	PP350809	47, 40, 64, 420	571	JQ977703.1 (100)	*carnica*
C	Q	C2y	PP350811	47, 40, 64, 420	571	JQ754650.1 (100)	*carnica*
A	P_0_Q	A1ha *	PP430326	47, 108, 482	637	KX463739.1 (99.83)	*iberiensis*
A	P_0_QQ	A4s	PP430327	47, 107, 192, 483	829	MW939597.1 (100)	n.a.
A	P_0_QQ	A4na *	PP430328	47, 108, 190, 482	827	KX463793.1 (99.75)	*iberiensis*

* Novel haplotype; n.a.—non available.

**Table 2:** Frequencies of detected *tRNA^leu^-cox2* haplotypes.

Haplotype	Sampled in Hives		Sampled on Flowers		Total	
	N	Frequency	N	Frequency	N	Frequency
C1a	72	0.4675	83	0.5390	155	0.5032
C2l	15	0.0974	10	0.0649	25	0.0812
C2e	34	0.2208	24	0.1558	58	0.1883
C2d	7	0.0455	5	0.0325	12	0.0390
C2c	19	0.1234	28	0.1818	47	0.1526
C2j	1	0.0065	0	0.0000	1	0.0032
C2s	4	0.0260	0	0.0000	4	0.0130
C2d7	0	0	1	0.0065	1	0.0032
C2i	0	0	1	0.0065	1	0.0032
C2y	0	0	1	0.0065	1	0.0032
A1ha	1	0.0065	0	0.0000	1	0.0032
A4s	1	0.0065	0	0.0000	1	0.0032
A4na	0	0	1	0.0065	1	0.0032
Total	154	1.0000	154	1.0000	308	1.0000

**Table 3:** Frequencies of detected *cox1* haplotypes.

Haplotype	Sampled in Hives		Sampled on Flowers		Total	
	N	Frequency	N	Frequency	N	Frequency
HpB01	37	0.2403	27	0.1753	64	0.2078
HpB02	68	0.4416	66	0.4286	134	0.4351
HpB03	24	0.1558	41	0.2662	65	0.2110
HpB04	14	0.0909	10	0.0649	24	0.0779
HpB05	3	0.0195	1	0.0065	4	0.0130
HpB06	0	0.0000	4	0.0260	4	0.0130
HpB07	3	0.0195	1	0.0065	4	0.0130
HpB08	0	0.0000	1	0.0065	1	0.0032
HpB09	0	0.0000	1	0.0065	1	0.0032
HpB10	0	0.0000	1	0.0065	1	0.0032
HpB11	1	0.0065	0	0.0000	1	0.0032
HpB12	1	0.0065	0	0.0000	1	0.0032
HpB13	1	0.0065	0	0.0000	1	0.0032
HpB14	1	0.0065	0	0.0000	1	0.0032
HpB15	1	0.0065	0	0.0000	1	0.0032
HpB16	0	0.0000	1	0.0065	1	0.0032
Total	154	1.0000	154	1.0000	308	1.0000

**Table 4:** Haplotype diversity parameters of *tRNA^leu^-cox2* and *cox1.*

Haplotype Sampling	N	*H*	*Hd*	*π*	*D*
*tRNA^leu^-cox2* (796 bp)					
In hives	154	9	0.70970	0.00201 (1.7583 × 10^−6^)	−3.17613 (0.00149)
On flowers	154	9	0.65096	0.00139 (1.02984 × 10^−6^)	−3.19736 (0.00139)
Total	308	13	0.68186	0.00172 (1.38726 × 10^−6^)	−3.02386 (0.00249)
*cox1* (658 bp)					
In hives	154	11	0.71845	0.00229 (2.35701 × 10^−6^)	−1.58841 (0.11219)
On flowers	154	11	0.71420	0.00177 (1.61582 × 10^−6^)	−1.56860 (0.11674)
Total	308	16	0.71866	0.00203 (1.96124 × 10^−6^)	−1.60658 (0.10815)

N number of individuals, *H* number of haplotypes, *Hd* haplotype diversity, *π* nucleotide diversity (variance), and *D* Tajima’s statistics (*p*-value under normal distributions).

## Data Availability

The sequence and annotation data presented in this study are publicly *available* in Genbank under accession numbers (Table 1 and PP401657–PP401672) and in Supplementary File S1 and File S3.

## Supplementary Materials

The following supporting information can be downloaded at https://www.mdpi.com/article/10.3390/insects15070495/s1, Table S1a: Distribution of *tRNA^leu^-cox2* haplotypes by region (counts), Table S1b: Distribution of *cox1* haplotypes by region (counts), Table S2: Combination of absolute frequencies of *cox1* and *tRNA^leu^-cox2* haplotypes, File S1: FASTA sequences of identified haplotypes *tRNA^leu^-cox2*, File S2: Sequence alignment of haplotypes in mitochondrial *tRNA^leu^-cox2* intergenic region in CLUSTAL format, File S3: FASTA sequences of identified haplotypes *cox1,* File S4: Sequence alignment of haplotypes in mitochondrial *cox1* region in CLUSTAL format.
