# Supplementary material for: Haplotype Diversity in mtDNA of Honeybee in the Czech Republic Confirms Complete Replacement of Autochthonous Population with the C Lineage
Source: Insects. 2024 Jul 2;15(7):495. doi: 10.3390/insects15070495 (PMC11276638; doi:10.3390/insects15070495)
Supplement: Supplementary file 1 [file insects-15-00495-s001.zip › File S2.pdf]

File S2. Sequence alignment of haplotypes in mitochondrial *tRNA^leu^-cox2* intergenic region in CLUSTAL format (Kalign 3.3.1)

Restriction site *Dra*I

*tRNA^leu^* – gray; P₀ – orange; Q – blue (2nd duplication of Q - black); *cox2* – purple

```
Site position by Franck et al. (2000)        1               a
Site position without P0 and Q insertion  25
C1a                 AACTTAAGATTCAAATATAAAGTATTTTTAAACTTTTATTAAAA----------------
C2l                 AACTTAAGATTCAAATATAAAGTATTTTTAAACTTTTATTAAAA----------------
C2e                 AACTTAAGATTCAAATATAAAGTATTTTTAAACTTTTATTAAAA----------------
C2d                 AACTTAAGATTCAAATATAAAGTATTTTTAAACTTTTATTAAAA----------------
C2c                 AACTTAAGATTCAAATATAAAGTATTTTTAAACTTTTATTAAAA----------------
C2j                 AACTTAAGATTCAAATATAAAGTATTTTTAAACTTTTATTAAAA----------------
C2s                 AACTTAAGATTCAAATATAAAGTATTTTTAAACTTTTATTAAAA----------------
C2d7                AACTTAAGATTCAAATATAAAGTA-TTTTAAACTTTTATTAAAA----------------
C2i                 AACTTAAGATTCAAATATAAAGTATTTTTAAACTTTTATTAAAA----------------
C2y                 AACTTAAGATTCAAATATAAAGTATTTTTAAACTTTTATTAAAA----------------
A1ha                AACTTAAGATTCAAATATAAAGTATTTTTAAACTTTTATTAAAATTAATAAATTAATATA
A4s                 AACTTAAGATTCAAATATAAAGTATTTTTAAACTTTTATTAAAATTAATAAATTAATATA
A4na                AACTTAAGATTCAAATATAAAGTATTTTTAAACTTTTATTAAAATTAATAAATTAATATA
Site position with P0 and Q insertion     25
```

```
                      b   2c  3 d   456 7             8     f      9  10 h
                                                                      45  48
C1a                 ---------------------------------------------------TTTCCCCAC
C2l                 ---------------------------------------------------TTT-CCCAC
C2e                 ---------------------------------------------------TTT-CCCAC
C2d                 ---------------------------------------------------TTT-CCCAC
C2c                 ---------------------------------------------------TTT-CCCAC
C2j                 ---------------------------------------------------TTT-CCCAC
C2s                 ---------------------------------------------------TTT-CCCAC
C2d7                ---------------------------------------------------TTT-CCCAC
C2i                 ---------------------------------------------------TTT-CCCAC
C2y                 ---------------------------------------------------TTT-CCCAC
A1ha                AAATAAAACAAAATATAACAAAATATATTTATTAAAATTTAATTTATTAAAATTCCCCAC
A4s                 AAATAAAACAAAATATAACAGAATATATTTATTAAAATTTAATTTATTAAAATT-CCCAC
A4na                AAATAAAACAAAATATAACAAAATATATTTATTAAAATTTAATTTATTAAAATTCCCCAC
                                        Gap37/81                          112 115
112 – parsimony informative site
```

```
                            11      12  13               14 j
                                                               90
C1a                 TTAATTCATATTAATTTAAAAATAAATTAATAACAATTTTTAATAAAATAAATAATTAAT
C2l                 TTAATTCATATTAATTTAAAAATAAATTAATAACAATTTTTAATAAAATAAATAATTAAT
C2e                 TTAATTCATATTAATTTAAAAATAAATTAATAACAA-TTTTAATAAAATAAATAATTAAT
C2d                 TTAATTCATATTAATTTAAAAATAAATTAATAACAATTTTTAATAAAATAAATAATTAAT
C2c                 TTAATTCATATTAATTTAAAAATAAATTAATAACAATTTTTAATAAAATAAATAATTAAT
C2j                 TTAATTCATATTAATTTAAAAATAAATTAATAACAATTTTTAATAAAATAAATAATTAAT
C2s                 TTAATTCATATTAATTTAAAAATAAATTAATAACAATTTTTAATAAAATAAATAATTAAT
C2d7                TTAATTCATATTAATTTAAAAATAAATTAATAACAATTTTTAATAAAATAAATAATTAAT
C2i                 TTAATTCATATTAATTTAAAAATAAATTAATAACAATTTTTAATAAAATAAATAATTAAT
C2y                 TTAATTCATATTAATTTAAAAATAAATTAATAACAATTTTTAATAAAATAAATAATTAAT
A1ha                TTAATTCATATTAATTTAAAAATAAATTAATAACAA-TTTTAATAAAATAAATAATTAAT
A4s                 TTAATTCATATTAATTTAAAAATAAATTAATAACAA-TTTTAATAAAATAAATAATTAAT
A4na                TTAATTCATATTAATTTAAAAATAAATTAATAACAA-TTTTAATAAAATAAATAATTAAT
                                                        161
```

```
                                         15  16
                     116               134 135
C1a                 TTTATTTTTATATTGAATTTTAAATTCAATCTTAAAGATTTAATCTTTTTATTAAAATTA
C2l                 TTTATTTTTATATTGAATTTTAAATTCAATCTTAAAGATTTAATCTTTTTATTAAAATTA
C2e                 TTTATTTTTATATTGAATTTTAAATTCAATCTTAAAGATTTAATCTTTTTATTAAAATTA
C2d                 TTTATTTTTATATTGAATTTTAAATTCAATCTTAAAGATTTAATCTTTTTATTAAAATTA
C2c                 TTTATTTTTATATTGAATTTTAAATTCAATCTTAAAGATTTAATCTTTTTATTAAAATTA
C2j                 TTTATTTTTATATTGAATTTTAAATTCAATCTTAAAGATTTAATCTTTTTATTAAAATTA
C2s                 TTTATTTTTATATTGAATTTAAAATTCAATCTTAAAGATTTAATCTTTTTATTAAAATTA
C2d7                TTTATTTTTATATTGAATTTTAAATTCAATCTTAAAGATTTAATCTTTTTATTAAAATTA
C2i                 TTTATTTTTATATTGAATTTTAAATTCAATCTTAAAGATTTAATCTTTTTATTAAAATTA
C2y                 TTTATTTTTATATTGAATTTTAAATTCAATCTTAAAGATTTAATCTTTTTATTAAAATTA
A1ha                TT-ATTTTTATATTGAATTTTTAATTCAATCTTAAAGATTTAATCTTTTTATTAAAATTA
A4s                 TTTATTTTTATATTGAATTTTTAATTCAATCTTAAAGATTTAATCTTTTTATTAAAATTA
A4na                TT-ATTTTTATATTGAATTTTTAATTCAATCTTAAAGATTTAATCTTTTTATTAAAATTA
                     183               201 202
201, 202 – parsimony informative site
```

```
                                       17         18  19              n 20
                              187 189            205 207              223
C1a                 ATAAATTAATATAAAATAAAACAAAATATAACAGAATATATTTATTAAAATTTAATTTAT
C2l                 ATAAATTAATATAAA-TAAAACAAAATATAACAGAATATATTTATTAAAATTTAATTTAT
C2e                 ATAAATTAATATAAAATAAAACAAAATATAACAGAATATATTTATTAAAATTTAATTTAT
C2d                 ATAAATTAATATAAAATAAAACAAAATATAACAGAATATATTTATTAAAATTTAATTTAT
C2c                 ATAAATTAATATAAAATAAAACAAAATATAACAGAATATATTTATTAAAATTTAATTTAT
C2j                 ATAAATTAATATATAATAAAACAAAATATAACAGAATATATTTATTAAAATTTAATTTAT
C2s                 ATAAATTAATATAAAATAAAACAAAATATAACAGAATATATTTATTAAAATTTAATTTAT
C2d7                ATAAATTAATATAAAATAAAACAAAATATAACAGAATATATTTATTAAAATTTAATTTAT
C2i                 ATAAATTAATATAAAATAAAACAAAATATAACAAAATATATTTATTAAAATTTAATTTAT
C2y                 ATAAATTAATATAAAATAAAACAAAATATAACAGAATATATTTATTAAA-TTTAATTTAT
A1ha                ATAAATTAATATAAAATAAAACAAAATATAATAGAATATATTTATTAAAATTTAATTTAT
A4s                 ATAAATTAATATAAAATAAAACAAAATATAACAGAATATATTTATTAAAATTTAATTTAT
A4na                ATAAATTAATATAAAATAAAACAAAATATAACAGAATATATTTATTAAAATTTAATTTAT
                              254 256            272 274              290
```

```
C1a             TAAA--------------------------------------------------------
C2l             TAAA--------------------------------------------------------
C2e             TAAA--------------------------------------------------------
C2d             TAAA--------------------------------------------------------
C2c             TAAA--------------------------------------------------------
C2j             TAAA--------------------------------------------------------
C2s             TAAA--------------------------------------------------------
C2d7            TAAA--------------------------------------------------------
C2i             TAAA--------------------------------------------------------
C2y             TAAA--------------------------------------------------------
A1ha            TAAA--------------------------------------------------------
A4s             TAAAATTCCCCACTTAATTCATATTAATTTAAAAATAAATTAATAACAATTTTAATAAAA
A4na            TAAAATT-CCCACTTAATTCATATTAATTTAAAAATAAATTAATAATAATTTTAATAAAA
                       Gap70/308                                Gap110/347

C1a             ------------------------------------------------------------
C2l             ------------------------------------------------------------
C2e             ------------------------------------------------------------
C2d             ------------------------------------------------------------
C2c             ------------------------------------------------------------
C2j             ------------------------------------------------------------
C2s             ------------------------------------------------------------
C2d7            ------------------------------------------------------------
C2i             ------------------------------------------------------------
C2y             ------------------------------------------------------------
A1ha            ------------------------------------------------------------
A4s             TAAATAATTAATTTTATTTTTATATTGAATTTTTAATTCAATCTTAAAGATTTAATCTTT
A4na            TAAATAATTAA-TTTATTTTTATATTGAATTTTTAATTCAATCTTAAAGATTTAATCTTT
                           Gap135/372

C1a             ------------------------------------------------------------
C2l             ------------------------------------------------------------
C2e             ------------------------------------------------------------
C2d             ------------------------------------------------------------
C2c             ------------------------------------------------------------
C2j             ------------------------------------------------------------
C2s             ------------------------------------------------------------
C2d7            ------------------------------------------------------------
C2i             ------------------------------------------------------------
C2y             ------------------------------------------------------------
A1ha            ------------------------------------------------------------
A4s             TTATTAAAATTAATAAATTAATATAAAATAAAACAAAATATAACAGAATATATTTATTAA
A4na            TTATTAAAATTAATAAATTAATATAAAATAAAACAAAATATAACAGAATATATTTATTAA

                                              21
                                             252
C1a             ----------------ATTTCCACATGATTTATATTTATATTTCAAGAATCAAATTCATA
C2l             ----------------ATTTCCACATGATTTATATTTATATTTCAAGAATCAAATTCATA
C2e             ----------------ATTTCCACATGATTCATATTTATATTTCAAGAATCAAATTCATA
C2d             ----------------ATTTCCACATGATTCATATTTATATTTCAAGAATCAAATTCATA
C2c             ----------------ATTTCCACATGATTTATATTTATATTTCAAGAATCAAATTCATA
C2j             ----------------ATTTCCACATGATTCATATTTATATTTCAAGAATCAAATTCATA
C2s             ----------------ATTTCCACATGATTCATATTTATATTTCAAGAATCAAATTCATA
C2d7            ----------------ATTTCCACATGATTCATATTTATATTTCAAGAATCAAATTCATA
C2i             ----------------ATTTCCACATGATTCATATTTATATTTCAAGAATCAAATTCATA
C2y             ----------------ATTTCCACATGATTTATATTTATATTTCAAGAATCAAATTCATA
A1ha            ----------------ATTTCCACATGATTCATATTTATATTTCAAGAATCAAATTCATA
A4s             AATTTAATTTATTAAAATTTCCACATGATTCATATTTATATTTCAAGAATCAAATTCATA
A4na            AATTTAATTTATTAAAATTTCCACATGATTCATATTTATATTTCAAGAATCAAATTCATA
                                             511
```

511 – parsimony informative site

```
                                                              22
                                                             327
C1a             TTATGCTGATAATTTAATTTCATTTCATAATATAGTTATAATAATTATTATTATAATTTC
C2l             TTATGCTGATAATTTAATTTCATTTCATAATATAGTTATAATAATTATTATTATAATTTC
C2e             TTATGCTGATAATTTAATTTCATTTCATAATATAGTTATAATAATTATTATTATAATTTC
C2d             TTATGCTGATAATTTAATTTCATTTCATAATATAGTTATAATAATTATTATTATAATTTC
C2c             TTATGCTGATAATTTAATTTCATTTCATAATATAGTTATAATAATTATTATTATAATTTC
C2j             TTATGCTGATAATTTAATTTCATTTCATAATATAGTTATAATAATTATTATTATAATTTC
C2s             TTATGCTGATAATTTAATTTCATTTCATAATATAGTTATAATAATTATTATTATAATTTC
C2d7            TTATGCTGATAATTTAATTTCATTTCATAATATAGTTATAATAATTATTATTATAATTTC
C2i             TTATGCTGATAATTTAATTTCATTTCATAATATAGTTATAATAATTATTATTATAATTTC
C2y             TTATGCTGATAATTTAATTTCATTTCATAATATAGTTATAATAATTATTATTATAATTTC
A1ha            TTATGCTGATAATTTAATTTCATTTCATAATATAGTTATAATAATCATTATTATAATTTC
A4s             TTATGCTGATAATTTAATTTCATTTCATAATATAGTTATAATAATCATTATTATAATTTC
A4na            TTATGCTGATAATTTAATTTCATTTCATAATATAGTTATAATAATCATTATTATAATTTC
                                                             586
```

586 – parsimony informative site

```
                                                       23     24     25
                                                      381    387
C1a             AACATTAACTGTATATATTATTTTAGATTTATTTATAAACAAATTCTCAAATTTATTTTT
C2l             AACATTAACTGTATATATTATTTTAGATTTATTTATAAACAAATTCTCAAATTTATTTTT
C2e             AACATTAACTGTATATATTATTTTAGATTTATTTATAAACAAATTTTCAAATTTATTTTT
C2d             AACATTAACTGTATATATTATTTTAGATTTATTTATAAACAAATTTTCAAATTTATTTTT
C2c             AACATTAACTGTATATATTATTTTAGATTTATTTATAAACAAATTCTCAAATTTATTTTT
C2j             AACATTAACTGTATATATTATTTTAGATTTATTTATAAACAAATTTTCAAATTTATTTTT
C2s             AACATTAACTGTATATATTATTTTAGATTTATTTATAAACAAATTTTCAAATTTATTTTT
C2d7            AACATTAACTGTATATATTATTTTAGATTTATTTATAAACAAATTTTCAAATTTATTTTT
C2i             AACATTAACTGTATATATTATTTTAGATTTATTTATAAACAAATTTTCAAATTTATTTTT
C2y             AACATTAACTGTATATATTATTTTAGATTTATTTATAAACAAATTCTCAAATTTATTTTT
A1ha            AACATTAACTGTATATATTATTTTAGATTTATTTATAAATAAATTTTCAAATTTATTTTT
A4s             AACATTAACTGTATATATTATTTTAGATTTATTTATAAATAAATTTTCAAATTTATTTTT
A4na            AACATTAACTGTATATATTATTTTAGATTTATTTATAAATAAATTTTCAAATTTATTTTT
                                                      640    646
```

640, 646 – parsimony informative site

```
                                                   26
                                                   436
C1a             ATTAAAAAATCATAATATTGAAATTATTTGAACAATTATTCCAATTATTATTCTATTAAT
C2l             ATTAAAAAATCATAATATTGAAATTATTTGAACAATTATTCCAATTATTATTCTATTAAT
C2e             ATTAAAAAATCATAATATTGAAATTATTTGAACAATTATTCCAATTATTATTCTATTAAT
C2d             ATTAAAAAATCATAATATTGAAATTATTTGAACAATTATTCCAATTATTATTCTATTAAT
C2c             ATTAAAAAATCATAATATTGAAATTATTTGAACAATTATTCCAATTATTATTCTATTAAT
C2j             ATTAAAAAATCATAATATTGAAATTATTTGAACAATTATTCCAATTATTATTCTATTAAT
C2s             ATTAAAAAATCATAATATTGAAATTATTTGAACAATTATTCCAATTATTATTCTATTAAT
C2d7            ATTAAAAAATCATAATATTGAAATTATTTGAACAATTATTCCAATTATTATTCTATTAAT
C2i             ATTAAAAAATCATAATATTGAAATTATTTGAACAATTATTCCAATTATTATTCTATTAAT
C2y             ATTAAAAAATCATAATATTGAAATTATTTGAACAATTATTCCAATTATTATTCTATTAAT
A1ha            ATTAAAAAATCATAATATTGAAATTATTTGAACAGTTATTCCAATTATTATTCTATTAAT
A4s             ATTAAAAAATCATAATATTGAAATTATTTGAACAGTTATTCCAATTATTATTCTATTAAT
A4na            ATTAAAAAATCATAATATTGAAATTATTTGAACAGTTATTCCAATTATTATTCTATTAAT
                                                  695
```

695 – parsimony informative site

```
                27
C1a             TATTTGTTTTCCATCATTAAAAATTTTATATTTAATTGATGAAATTGTAAATCCTTTTTT
C2l             TATTTGTTTTCCATCATTAAAAATTTTATATTTAATTGATGAAATTGTAAATCCTTTTTT
C2e             TATTTGTTTTCCATCATTAAAAATTTTATATTTAATTGATGAAATTGTAAATCCTTTTTT
C2d             TATTTGTTTTCCATCATTAAAAATTTTATATTTAATTGATGAAATTGTAAATCCTTTTTT
C2c             TATTTGTTTTCCATCATTAAAAATTTTATATTTAATTGATGAAATTGTAAATCCTTTTTT
C2j             TATTTGTTTTCCATCATTAAAAATTTTATATTTAATTGATGAAATTGTAAATCCTTTTTT
C2s             TATTTGTTTTCCATCATTAAAAATTTTATATTTAATTGATGAAATTGTAAATCCTTTTTT
C2d7            TATTTGTTTTCCATCATTAAAAATTTTATATTTAATTGATGAAATTGTAAATCCTTTTTT
C2i             TATTTGTTTTCCATCATTAAAAATTTTATATTTAATTGATGAAATTGTAAATCCTTTTTT
C2y             TATTTGTTTTCCATCATTAAAAATTTTATATTTAATTGATGAAATTGTAAATCCTTTTTT
A1ha            TATTTGTTTTCCATCATTAAAAATTTTATATTTAATTGATGAAATTGTAAATCCTTTTTT
A4s             TATTTGTTTTCCATCATTAAAAATTTTATATTTAATTGATGAAATTGTAAATCCTTTTTT
A4na            TATTTGTTTTCCATCATTAAAAATTTTATATTTAATTGATGAAATTGTAAATCCTTTTTT

C1a             TTCAATTAAATCAATT
C2l             TTCAATTAAATCAATT
C2e             TTCAATTAAATCAATT
C2d             TTCAATTAAATCAATT
C2c             TTCAATTAAATCAATT
C2j             TTCAATTAAATCAATT
C2s             TTCAATTAAATCAATT
C2d7            TTCAATTAAATCAATT
C2i             TTCAATTAAATCAATT
C2y             TTCAATTAAATCAATT
A1ha            TTCAATTAAATCAATT
A4s             TTCAATTAAATCAATT
A4na            TTCAATTAAATCAATT
```
