# Supplementary material for: Haplotype Diversity in mtDNA of Honeybee in the Czech Republic Confirms Complete Replacement of Autochthonous Population with the C Lineage
Source: Insects. 2024 Jul 2;15(7):495. doi: 10.3390/insects15070495 (PMC11276638; doi:10.3390/insects15070495)
Supplement: Supplementary file 1 [file insects-15-00495-s001.zip › File S4.pdf]

File S4. Sequence alignment of haplotypes in mitochondrial *cox1* region in CLUSTAL format (Kalign 3.3.1)

```
           1                                                  52
HpB01      GATCTTGTATATTATTCTAGCTTTATGATCTGGAATACTAGGATCATCAATGAGACTTAT
HpB02      GATCTTGTATATTATTCTAGCTTTATGATCTGGAATACTAGGATCATCAATGAGACTTAT
HpB03      GATCTTGTATATTATTCTAGCTTTATGATCTGGAATACTAGGATCATCAATGAGACTTAT
HpB04      GATCTTGTATATTATTCTAGCTTTATGATCTGGAATACTAGGATCATCAATGAGACTTAT
HpB05      GATCTTGTATATTATTCTAGCTTTATGATCTGGAATACTAGGATCATCAATGAGACTTAT
HpB06      GATCTTGTATATTATTCTAGCTTTATGATCTGGAATACTAGGATCATCAATGAGACTTAT
HpB07      AATCTTGTATATTATTCTAGCTTTATGATCTGGAATACTAGGATCATCAATGAGACTTAT
HpB08      GATCTTGTATATTATTCTAGCTTTATGATCTGGAATACTAGGATCATCAATAAGACTTAT
HpB09      GATCTTGTATATTATTCTAGCTTTATGATCTGGAATACTAGGATCATCAATGAGACTTAT
HpB10      GATCTTGTATATTATTCTAGCTTTATGATCTGGAATACTAGGATCATCAATGAGACTTAT
HpB11      GATCTTGTATATTATTCTAGCTTTATGATCTGGAATACTAGGATCATCAATGAGACTTAT
HpB12      AATCTTGTATATTATTCTAGCTTTATGATCTGGAATACTAGGATCATCAATGAGACTTAT
HpB13      GATCTTGTATATTATTCTAGCTTTATGATCTGGAATACTAGGATCATCAATGAGACTTAT
HpB14      GATCTTGTATATTATTCTAGCTTTATGATCTGGAATACTAGGATCATCAATGAGACTTAT
HpB15      GATCTTGTATATTATTCTAGCTTTATGATCTGGAATACTAGGATCATCAATGAGACTTAT
HpB16      GATCTTGTATATTATTCTAGCTTTATGATCTGGAATACTAGGATCATCAATGAGACTTAT
1 – parsimony informative site

                                                99 100
HpB01      TATTCGAATAGAATTAAGATCCCCAGGATCATGAATTAACAATGATCAAATTTATAATAC
HpB02      TATTCGAATAGAATTAAGATCCCCAGGATCATGAATTAGCAATGATCAAATTTATAATAC
HpB03      TATTCGAATAGAATTAAGATCCCCAGGATCATGAATTAGCAATGATCAAATTTATAATAC
HpB04      TATTCGAATAGAATTAAGATCCCCAGGATCATGAATTAGCAATGATCAAATTTATAATAC
HpB05      TATTCGAATAGAATTAAGATCCCCAGGATCATGAATTAACAATGATCAAATTTATAATAC
HpB06      TATTCGAATAGAATTAAGATCCCCAGGATCATGAATTAGCAATGATCAAATTTATAATAC
HpB07      TATTCGAATAGAATTAAGATCCCCAGGATCATGAATTAACAATGATCAAATTTATAATAC
HpB08      TATTCGAATAGAATTAAGATCCCCAGGATCATGAATTAACAATGATCAAATTTATAATAC
HpB09      TATTCGAATAGAATTAAGATCCCCAGGATCATGAATTAACAATGATCAAATTTATAATAC
HpB10      TATTCGAATAGAATTAAGATCCCCAGGATCATGAATTAGCAATGATCAAATTTATAATAC
HpB11      TATTCGAATAGAATTAAGATCCCCAGGATCATGAATTAGCAATGATCAAATTTATAATAC
HpB12      TATTCGAATAGAATTAAGATCCCCAGGATCATGAATTAGCAATGATCAAATTTATAATAC
HpB13      TATTCGAATAGAATTAAGATCCCCAGGATCATGAATTAACAATGATCAAATTTATAATAC
HpB14      TATTCGAATAGAATTAAGATCCCCAGGATCATGAATTAATAATGATCAAATTTATAATAC
HpB15      TATTCGAATAGAATTAAGATCCCCAGGATCATGAATTAATAATGATCAAATTTATAATAC
HpB16      TATTCGAATAGAATTAAGATCCCCAGGATCATGAATTAATAATGATCAAATTTATAATAC
99, 100 – parsimony informative site

                        133      142
HpB01      AATTGTTACTAGTCATGCATTCCTAATAATTTTTTTTATAGTTATACCATTTTTAATTGG
HpB02      AATTGTTACTAGTCATGCATTCCTAATAATTTTTTTTATAGTTATACCATTTTTAATTGG
HpB03      AATTGTTACTAGTCATGCATTCCTAATAATTTTTTTTATAGTTATACCATTTTTAATTGG
HpB04      AATTGTTACTAGTCATGCATTCCTAATAATTTTTTTTATAGTTATACCATTTTTAATTGG
HpB05      AATTGTTACTAGTCATGCATTCCTAATAATTTTTTTTATAGTTATACCATTTTTAATTGG
HpB06      AATTGTTACTAGTCATGCATTTCTAATAATTTTTTTTATAGTTATACCATTTTTAATTGG
HpB07      AATTGTTACTAGTCATGCATTCCTAATAATTTTTTTTATAGTTATACCATTTTTAATTGG
HpB08      AATTGTTACTAGTCATGCATTCCTAATAATTTTTTTTATAGTTATACCATTTTTAATTGG
HpB09      AATTGTTACTAGTCATGCATTCCTAATAATTTTTTTTATAGTTATACCATTTTTAATTGG
HpB10      AATTGTTACTAGTCATGCATTCCTAATAATTTTTTTTATAGTTATACCATTTTTAATTGG
HpB11      AATTGTTACTAGTCATGCATTCCTAATAATTTTTTTTATAGTTATACCATTTTTAATTGG
HpB12      AATTGTTACTAGTCATGCATTCCTAATAATTTTTTTTATAGTTATACCATTTTTAATTGG
HpB13      AATTGTTACTAGTCATGCATTCCTAATAATTTTTTTTATAGTTATACCATTTTTAATTGG
HpB14      AATTGTTACTAGCCATGCATTTCTAATAATTTTTTTTATAGTTATACCATTTTTAATTGG
HpB15      AATTGTTACTAGCCATGCATTTCTAATAATTTTTTTTATAGTTATACCATTTTTAATTGG
HpB16      AATTGTTACTAGCCATGCATTTCTAATAATTTTTTTTATAGTTATACCATTTTTAATTGG
133, 142 – parsimony informative site

                           196                                    235
HpB01      AGGATTTGGAAATTGGCTTATTCCTTTAATACTAGGATCACCTGATATAGCATTCCCCCG
HpB02      AGGATTTGGAAATTGGCTTATTCCTTTAATACTAGGATCACCTGATATAGCATTCCCCCG
HpB03      AGGATTTGGAAATTGGCTTATTCCTTTAATACTAGGATCACCTGATATAGCATTCCCCCG
HpB04      AGGATTTGGAAATTGGCTTATTCCTTTAATACTAGGATCACCTGATATAGCATTCCCCCG
HpB05      AGGATTTGGAAATTGGCTTATTCCTTTAATACTAGGATCACCTGATATAGCATTCCCCCG
HpB06      AGGATTTGGAAATTGGCTTATTCCTTTAATACTAGGATCACCTGATATAGCATTCCCCCG
HpB07      AGGATTTGGAAATTGGCTTATTCCTTTAATACTAGGATCACCTGATATAGCATTCCCCCG
HpB08      AGGATTTGGAAATTGGCTTATTCCTTTAATACTAGGATCACCTGATATAGCATTCCCCCG
HpB09      AGGATTTGGAAATTGGCTTATTCCTTTAATACTAGGATCACCTGATATAGCATTCCCCCG
HpB10      AGGATTTGGAAATTGACTTATTCCTTTAATACTAGGATCACCTGATATAGCATTCCCCCG
HpB11      AGGATTTGGAAATTGGCTTATTCCTTTAATACTAGGATCACCTGATATAGCATTCCCCCG
HpB12      AGGATTTGGAAATTGGCTTATTCCTTTAATACTAGGATCACCTGATATAGCATTCCCCCG
HpB13      AGGATTTGGAAATTGACTTATTCCTTTAATACTAGGATCACCTGATATAGCATTCCCCCG
HpB14      AGGATTTGGAAATTGGCTTATTCCTTTAATACTAGGATCACCTGATATAGCATTTCCCCG
HpB15      AGGATTTGGAAATTGGCTTATTCCTTTAATACTAGGATCACCTGATATAGCATTTCCCCG
HpB16      AGGATTTGGAAATTGGCTTATTCCTTTAATACTAGGATCACCTGATATAGCATTTCCCCG
196, 235 – parsimony informative site

                               259            271 274           285
HpB01      AATAAATAATATTAGATTTTGATTACTTCCTCCCTCATTATTTATACTTTTATTAAGAAA
HpB02      AATAAATAATATTAGATTTTGATTACTTCCTCCATCATTATTTATACTTTTATTAAGAAA
HpB03      AATAAATAATATTAGATTTTGATTACTTCCTCCCTCATTATTTATACTTTTATTAAGAAA
HpB04      AATAAATAATATTAGATTTTGATTACTTCCTCCCTCATTATTTATACTTTTATTAAGAAA
HpB05      AATAAATAATATTAGATTTTGATTACTTCCTCCCTCATTATTTATACTTTTATTAAGAAA
HpB06      AATAAATAATATTAGATTTTGATTACTTCCTCCCTCATTATTTATACTTTTATTAAGAAA
HpB07      AATAAATAATATTAGATTTTGATTACTTCCTCCCTCATTATTTATACTTTTATTAAGAAA
HpB08      AATAAATAATATTAGATTTTGATTACTTCCTCCCTCATTATTTATACTTTTATTAAGAAA
HpB09      AATAAATAATATTAGATTCTGATTACTTCCTCCCTCATTATTTATACTTTTATTAAGAAA
HpB10      AATAAATAATATTAGATTTTGATTACTTCCTCCCTCATTATTTATACTTTTATTAAGAAA
HpB11      AATAAATAATATTAGATTTTGATTACTTCCACCCTCATTATTTATACTTTTATTAAGAAA
HpB12      AATAAATAATATTAGATTTTGATTACTTCCTCCCTCATTATTTATACTTTTATTAAGAAA
HpB13      AATAAATAATATTAGATTTTGATTACTTCCTCCCTCATTATTTATACTTTTATTAAGAAA
HpB14      AATAAATAATATTAGATTTTGATTACTTCCTCCCTCATTATTTATACTTTTATTAAGAAA
HpB15      AATAAATAATATTAGATTTTGATTACTTCCTCCCTCATTATTTACACTTTTATTAAGAAA
HpB16      AATAAATAATATTAGATTTTGATTACTTCCTCCCTCATTATTTATACTTTTATTAAGAAA
```

```
                  310
HpB01    TTTATTTTATCCAAGACCAGGAACTGGATGAACAGTATATCCACCATTATCAGCATATTT
HpB02    TTTATTTTATCCAAGACCAGGAACTGGATGAACAGTATATCCACCATTATCAGCATATTT
HpB03    TTTATTTTATCCAAGACCAGGAACTGGATGAACAGTATATCCACCATTATCAGCATATTT
HpB04    TTTATTTTATCCAAGACCAGGAACTGGATGAACAGTATATCCACCATTATCAGCATATTT
HpB05    TTTATTTTATCCAAGACCAGGAACTGGATGAACAGTATATCCACCATTATCAGCATATTT
HpB06    TTTATTTTATCCAAGACCAGGAACTGGATGAACAGTATATCCACCATTATCAGCATATTT
HpB07    TTTATTTTATCCAAGACCAGGAACTGGATGAACAGTATATCCACCATTATCAGCATATTT
HpB08    TTTATTTTATCCAAGACCAGGAACTGGATGAACAGTATATCCACCATTATCAGCATATTT
HpB09    TTTATTTTATCCAAGACCAGGAACTGGATGAACAGTATATCCACCATTATCAGCATATTT
HpB10    TTTATTTTATCCAAGACCAGGAACTGGATGAACAGTATATCCACCATTATCAGCATATTT
HpB11    TTTATTTTATCCAAGACCAGGAACTGGATGAACAGTATATCCACCATTATCAGCATATTT
HpB12    TTTATTTTATCCAAGACCAGGAACTGGATGAACAGTATATCCACCATTATCAGCATATTT
HpB13    TTTATTTTATCCAAGACCAGGAACTGGATGAACAGTATATCCACCATTATCAGCATATTT
HpB14    TTTATTTTACCCAAGACCAGGAACTGGATGAACAGTATATCCACCATTATCAGCATATTT
HpB15    TTTATTTTACCCAAGACCAGGAACTGGATGAACAGTATATCCACCATTATCAGCATATTT
HpB16    TTTATTTTACCCAAGACCAGGAACTGGATGAACAGTATATCCACCATTATCAGCATATTT
310 - parsimony informative site
```

```
                                      389
HpB01    ATATCATTCTTCACCTTCAGTAGATTTTGCAATTTTTTCTCTTCATATATCAGGAATTTC
HpB02    ATATCATTCTTCACCTTCAGTAGATTTTGCAATTTTTTCTCTTCATATATCAGGAATTTC
HpB03    ATATCATTCTTCACCTTCAGTAGATTTTGCAATTTTTTCTCTTCATATATCAGGAATTTC
HpB04    ATATCATTCTTCACCTTCAGTAGATTTTGCAATTTTTTCTCTTCATATATCAGGAATTTC
HpB05    ATATCATTCTTCACCTTCAGTAGATTTTGCAATTTTTTCTCTTCATATATCAGGAATTTC
HpB06    ATATCATTCTTCACCTTCAGTAGATTTTGCAATTTTTTCTCTTCATATATCAGGAATTTC
HpB07    ATATCATTCTTCACCTTCAGTAGATTTTGCAATTTTTTCTCTTCATATATCAGGAATTTC
HpB08    ATATCATTCTTCACCTTCAGTAGATTTTGCAATTTTTTCTCTTCATATATCAGGAATTTC
HpB09    ATATCATTCTTCACCTTCAGTAGATTTTGCAATTTTTTCTCTTCATATATCAGGAATTTC
HpB10    ATATCATTCTTCACCTTCAGTAGATTTTGCAATTTTTTCTCTTCATATATCAGGAATTTC
HpB11    ATATCATTCTTCACCTTCAGTAGATTTTGCAATTTTTTCTCTTCATATATCAGGAATTTC
HpB12    ATATCATTCTTCACCTTCAGTAGATTTTGCAATTTTTTCTCTTCATATATCAGGAATTTC
HpB13    ATATCATTCTTCACCTTCAGTAGATTTTGCAATTTTTTCTCTTCATATATCAGGAATTTC
HpB14    ATATCATTCTTCACCTTCAGTAGATTTTGCAATTTTTTCTCTTCATATATCAGGAATTTC
HpB15    ATATCATTCTTCACCTTCAGTAGATTTTACAATTTTTTCTCTTCATATATCAGGAATTTC
HpB16    ATATCATTCTTCACCTTCAGTAGATTTTGCAATTTTTTCTCTTCATATATCAGGAATTTC
```

```
         421             437  442   448
HpB01    CTCAATTATAGGATCATTAAACTTAATAGTTACAATTATAATAATAAAAAATTTTTCTAT
HpB02    CTCAATTATAGGATCATTAAACTTAATAGTTACAATTATAATAATAAAAAATTTTTCTAT
HpB03    CTCAATTATAGGATCATTAAACTTAATAGTTACAATTATAATAATAAAAAATTTTTCTAT
HpB04    CTCAATTATAGGATCATTAAACTTAATGGTTACAATTATAATAATAAAAAATTTTTCTAT
HpB05    ATCAATTATAGGATCATTAAACTTAATAGTTACAATTATAATAATAAAAAATTTTTCTAT
HpB06    CTCAATTATAGGATCATTAAACTTAATAGTTACAATTATAATAATAAAAAATTTTTCTAT
HpB07    CTCAATTATAGGATCATTAAACTTAATAGTTACAATTATAATAATAAAAAATTTTTCTAT
HpB08    CTCAATTATAGGATCATTAAACTTAATAGTTACAATTATAATAATAAAAAATTTTTCTAT
HpB09    CTCAATTATAGGATCATTAAACTTAATAGTTACAATTATAATAATAAAAAATTTTTCTAT
HpB10    CTCAATTATAGGATCATTAAACTTAATAGTTACAATTATAATAATAAAAAATTTTTCTAT
HpB11    CTCAATTATAGGATCATTAAACTTAATAGTTACAATTATAATAATAAAAAATTTTTCTAT
HpB12    CTCAATTATAGGATCATTAAACTTAATGGTTACAATTATAATAATAAAAAATTTTTCTAT
HpB13    CTCAATTATAGGATCATTAAACTTAATAGTTACAATTATAATAATAAAAAATTTTTCTAT
HpB14    CTCAATTATAGGATCACTAAACTTAATAGTTACAATTATAATAATAAAAAATTTTTCTAT
HpB15    CTCAATTATAGGATCATTAAATTTAATAGTTACAATTATAATAATAAAAAATTTTTCTAT
HpB16    CTCAATTATAGGATCATTAAACTTAATAGTTACAATTATAATAATAAAAAATTTTTCTAT
448 - parsimony informative site
```

```
HpB01    AAATTATGACCAAATTTCATTATTTCCATGATCAGTTTTTATTACAGCAATTTTATTAAT
HpB02    AAATTATGACCAAATTTCATTATTTCCATGATCAGTTTTTATTACAGCAATTTTATTAAT
HpB03    AAATTATGACCAAATTTCATTATTTCCATGATCAGTTTTTATTACAGCAATTTTATTAAT
HpB04    AAATTATGACCAAATTTCATTATTTCCATGATCAGTTTTTATTACAGCAATTTTATTAAT
HpB05    AAATTATGACCAAATTTCATTATTTCCATGATCAGTTTTTATTACAGCAATTTTATTAAT
HpB06    AAATTATGACCAAATTTCATTATTTCCATGATCAGTTTTTATTACAGCAATTTTATTAAT
HpB07    AAATTATGACCAAATTTCATTATTTCCATGATCAGTTTTTATTACAGCAATTTTATTAAT
HpB08    AAATTATGACCAAATTTCATTATTTCCATGATCAGTTTTTATTACAGCAATTTTATTAAT
HpB09    AAATTATGACCAAATTTCATTATTTCCATGATCAGTTTTTATTACAGCAATTTTATTAAT
HpB10    AAATTATGACCAAATTTCATTATTTCCATGATCAGTTTTTATTACAGCAATTTTATTAAT
HpB11    AAATTATGACCAAATTTCATTATTTCCATGATCAGTTTTTATTACAGCAATTTTATTAAT
HpB12    AAATTATGACCAAATTTCATTATTTCCATGATCAGTTTTTATTACAGCAATTTTATTAAT
HpB13    AAATTATGACCAAATTTCATTATTTCCATGATCAGTTTTTATTACAGCAATTTTATTAAT
HpB14    AAATTATGACCAAATTTCATTATTTCCATGATCAGTTTTTATTACAGCAATTTTATTAAT
HpB15    AAATTATGACCAAATTTCATTATTTCCATGATCAGTTTTTATTACAGCAATTTTATTAAT
HpB16    AAATTATGACCAAATTTCATTATTTCCATGATCAGTTTTTATTACAGCAATTTTATTAAT
```

```
                548                   571
HpB01    TATATCATTACCTGTATTAGCTGGAGCAATTACTATACTATTATTTGATCGAAATTTTAA
HpB02    TATATCATTACCTGTATTAGCTGGAGCAATTACTATACTATTATTTGATCGAAATTTTAA
HpB03    TATATCATTACCTGTATTAGCTGGAGCAATTACTATACTATTATTTGATCGAAATTTTAA
HpB04    TATATCATTACCTGTATTAGCTGGAGCAATTACTATACTATTATTTGATCGAAATTTTAA
HpB05    TATATCATTACCTGTATTAGCTGGAGCAATCACTATACTATTATTTGATCGAAATTTTAA
HpB06    TATATCATTACCTGTATTAGCTGGAGCAATTACTATACTATTATTTGATCGAAATTTTAA
HpB07    TATATCATTACCTGTATTAGCTGGAGCAATTACTATACTATTATTTGATCGAAATTTTAA
HpB08    TATATCATTACCTGTATTAGCTGGAGCAATTACTATACTATTATTTGATCGAAATTTTAA
HpB09    TATATCATTACCTGTATTAGCTGGAGCAATTACTATACTATTATTTGATCGAAATTTTAA
HpB10    TATATCATTACCTGTATTAGCTGGAGCAATTACTATACTATTATTTGATCGAAATTTTAA
HpB11    TATATCATTACCTGTATTAGCTGGAGCAATTACTATACTATTATTTGATCGAAATTTTAA
HpB12    TATATCATTACCTGTATTAGCTGGAGCAATTACTATACTATTATTTGATCGAAATTTTAA
HpB13    TATATCATTACCTGTATTAGCTGGAGCAATTACTATACTATTATTTGATCGAAATTTTAA
HpB14    TATATCACTACCTGTATTAGCTGGAGCAATTACTATACTATTATTTGATCGAAATTTTAA
HpB15    TATATCACTACCTGTATTAGCTGGAGCAATTACTATACTATTATTTGATCGAAATTTTAA
HpB16    TATATCATTACCTGTATTAGCTGGAGCAATTACTATACTATTATTTGATCGAAATTTTAA
548 - parsimony informative site
```

```
                                                      641 643
HpB01      TACATCATTTTTCGATCCTATAGGAGGTGGAGATCCAATTCTTTATCAACATTTATTT
HpB02      TACATCATTTTTCGATCCTATAGGAGGTGGAGATCCAATTCTTTATCAACATTTATTT
HpB03      TACATCATTTTTCGATCCTATAGGAGGTGGAGATCCAATTCTTTATCAACATTTATTT
HpB04      TACATCATTTTTCGATCCTATAGGAGGTGGAGATCCAATTCTTTATCAACATTTATTT
HpB05      TACATCATTTTTCGATCCTATAGGAGGTGGAGATCCAATTCTTTATCAACATTTATTT
HpB05      TACATCATTTTTCGATCCTATAGGAGGTGGAGATCCAATTCTTTATCAACATTTATTT
HpB06      TACATCATTTTTCGATCCTATAGGAGGTGGAGATCCAATTCTTTATCAACATTTATTT
HpB07      TACATCATTTTTCGATCCTATAGGAGGTGGAGATCCAATTCTTTATCAACATTTATTT
HpB08      TACATCATTTTTCGATCCTATAGGAGGTGGAGATCCAATTCTTTATCAACATTTATTT
HpB09      TACATCATTTTTCGATCCTATAGGAGGTGGAGATCCAATTCTTTATCAACATTTATTT
HpB10      TACATCATTTTTCGATCCTATAGGAGGTGGAGATCCAATTCTTTATCAACATTTATTT
HpB11      TACATCATTTTTCGATCCTATAGGAGGTGGAGATCCAATTCTTTATCAACATTTATTT
HpB12      TACATCATTTTTCGATCCTATAGGAGGTGGAGATCCAATTCTTTATCAACATTTATTT
HpB13      TACATCATTTTTCGATCCTATAGGAGGTGGAGATCCAATTCTTTATCAACATTTATTT
HpB14      TACATCATTTTTCGATCCTATAGGAGGTGGAGATCCAATTTTATATCAACATTTATTT
HpB15      TACATCATTTTTCGATCCTATAGGAGGTGGAGATCCAATTTTATATCAACATTTATTT
HpB16      TACATCATTTTTCGATCCTATAGGAGGTGGAGATCCAATTTTATATCAACATTTATTT
```

641, 643 - parsimony informative site

Sites with alignment gaps or missing data: 0

Invariable (monomorphic) sites: 638

Variable (polymorphic) sites: 20 (Total number of mutations: 20)

Singleton variable sites: 8

Parsimony informative sites: 12

Singleton variable sites (two variants): 8

Site positions: 52 259 271 274 285 389 437 442

Parsimony informative sites (two variants): 12

Site positions: 1 99 100 133 142 196 235 310 421 448 548 571

Variable sites (three variants): 0

Variable sites (four variants): 0

Protein Coding Region assignation: No

Number of polymorphic (segregating) sites, S: 20

Total number of mutations, Eta: 20

Number of Haplotypes, h: 16

Haplotype (gene) diversity, Hd: 0,993

Variance of Haplotype diversity: 0,00053

Standard Deviation of Haplotype diversity: 0,023

Nucleotide diversity, Pi: 0,00668

Sampling variance of Pi: 0,0000015

Standard deviation of Pi: 0,00124

Nucleotide diversity (Jukes and Cantor), Pi(JC): 0,00673

Theta (per site) from Eta: 0,00899

Theta (per site) from S, Theta-W: 0,00899

Variance of theta (no recombination): 0,0000134

Standard deviation of theta (no recombination): 0,00366

Variance of theta (free recombination): 0,0000040

Standard deviation of theta (free recombination): 0,00201

Finite Sites Model

Theta (per site) from Pi: 0,00674

Theta (per site) from S: 0,00916

Theta (per site) from Eta: 0,00908

Average number of nucleotide differences, k: 4,397

Stochastic variance of k (no recombination), Vst(k): 4,604

Sampling variance of k (no recombination), Vs(k): 0,610

Total variance of k (no recombination), V(k): 5,214

Stochastic variance of k (free recombination), Vst(k): 1,466

Sampling variance of k (free recombination), Vs(k): 0,183

Total variance of k (free recombination), V(k): 1,649

Theta (per sequence) from S, Theta-W: 5,916

Variance of theta (no recombination): 5,798

Variance of theta (free recombination): 1,750

==== Analysis using the total number of mutations ====

Number of polymorphic (segregating) sites, S: 20

Total number of mutations, Eta: 20

Total number of singleton mutations, Eta(s): 8

Average number of pairwise nucleotide differences, k: 4,397

Nucleotide diversity, Pi: 0,00668

Theta estimated from k: 4,397

Theta estimated from Eta(s): 7,529

Theta estimated from Eta: 5,916

Number of Haplotypes, h: 16

Haplotype (gene) diversity, Hd: 0,993

Variance of Haplotype diversity: 0,00053

Fu's Fs statistic: -12,587

Strobeck's S statistic: 1,000

(Probability that NHap <= 16)

Probability that [NHap = 16]: 0,000

==== Analysis using only biallelic positions ====

Number of segregating sites analyzed, S: 20

Fu and Li's D* test statistic, FLD*: -0,42753

Fu and Li's F* test statistic, FLF*: -0,62780

Achaz Y* test statistic, AY*: -1,28512

=== Results from DnaSP v5 ===

Fu and Li's D* test statistic: -0,42753

Statistical significance: Not significant, P > 0.10

Fu and Li's F* test statistic: -0,68708

Statistical significance: Not significant, P > 0.10

Total number of sites (excluding sites with gaps / missing data): 658

Number of polymorphic (segregating) sites, S: 20

Total number of mutations, Eta: 20

Average number of nucleotide differences, k: 4,39706

Nucleotide diversity, Pi: 0,00668

Theta (per sequence) from Eta: 5,91588

Theta (per site) from Eta: 0,00899

Tajima's D: -1,01604   Statistical significance: Not significant, P > 0.10

```
============ Synonymous and NonSynonymous Substitutions ============

No coding region defined

Calculated using the total number of mutations
```
