# Supplementary material for: Haplotype Diversity in mtDNA of Honeybee in the Czech Republic Confirms Complete Replacement of Autochthonous Population with the C Lineage
Source: Insects. 2024 Jul 2;15(7):495. doi: 10.3390/insects15070495 (PMC11276638; doi:10.3390/insects15070495)
Supplement: Supplementary file 1 [file insects-15-00495-s001.zip › Table S1a and S1b.pdf]

Table S1a. Distribution of *$tRNA^{leu}$-cox2* haplotypes by Region (counts)

	$tRNA^{leu}$-cox2 **haplotypes**													
**Region**	**C1a**	**C2l**	**C2e**	**C2d**	**C2c**	**C2j**	**C2s**	**C2d7**	**C2i**	**C2y**	**A1ha**	**A4s**	**A4na**	**Total**
HKK	9	1	6	1	5									22
JHC	13	2	5	3	3		1				1			28
JHM	21	4	1		3				1					30
KVK	5	1	3		1									10
LBK	7	1	3		4								1	16
MSK	13	4	2	1	3		1							24
OLK	11		3	2	4									20
PAK	11	1	1		3									16
PHA	1		1		2									4
PLK	14		9	2	2	1								28
STC	23	5	10		7			1		1		1		48
ULK	12	2	7		6		1							28
VYS	9	3	5	2	1									20
ZLK	6	1	2	1	3		1							14
**Total**	155	25	58	12	47	1	4	1	1	1	1	1	1	308
	50,3%	8,1%	18,8%	3,9%	15,3%	0,3%	1,3%	0,3%	0,3%	0,3%	0,3%	0,3%	0,3%	100,0%

Note: Region code: HKK (Hradec Králové Region), JHC (South Bohemia Region), JHM (South Moravia Region), KVK (Karlovy Vary Region), LBK (Liberec Region), MSK (Moravian-Silesian Region), OLK (Olomouc Region), PAK (Pardubice Region), PHA (Capital City of Prague), PLK (Plzeň Region), STC (Central Bohemia Region), ULK (Ústí Region), VYS (Vysočina Region), ZLK (Zlín Region)

Table S1b. Distribution of *cox1* haplotypes by Region (counts)

	***cox1* haplotypes**																
**Region**	**HpB01**	**HpB02**	**HpB03**	**HpB04**	**HpB05**	**HpB06**	**HpB07**	**HpB08**	**HpB09**	**HpB10**	**HpB11**	**HpB12**	**HpB13**	**HpB14**	**HpB15**	**HpB16**	**Total**
HKK	7	8	6	1													22
JHC	6	14	1	1		1	2	1			1			1			28
JHM	2	18	6	3								1					30
KVK	3	4	2	1													10
LBK	3	5	6	1												1	16
MSK	2	13	2	5	1		1										24
OLK	3	7	6		2	2											20
PAK	1	10	4	1													16
PHA		2	2														4
PLK	12	12	4														28
STC	9	20	10	5			1		1	1					1		48
ULK	6	11	7	2		1							1				28
VYS	7	5	5	3													20
ZLK	3	5	4	1	1												14
Total	64	134	65	24	4	4	4	1	1	1	1	1	1	1	1	1	**308**
	20,8%	43,5%	21,1%	7,8%	1,3%	1,3%	1,3%	0,3%	0,3%	0,3%	0,3%	0,3%	0,3%	0,3%	0,3%	0,3%	100,0%

Note: Region code: HKK (Hradec Králové Region), JHC (South Bohemia Region), JHM (South Moravia Region), KVK (Karlovy Vary Region), LBK (Liberec Region), MSK (Moravian-Silesian Region), OLK (Olomouc Region), PAK (Pardubice Region), PHA (Capital City of Prague), PLK (Plzeň Region), STC (Central Bohemia Region), ULK (Ústí Region), VYS (Vysočina Region), ZLK (Zlín Region)
