# Supplementary material for: Haplotype Diversity in mtDNA of Honeybee in the Czech Republic Confirms Complete Replacement of Autochthonous Population with the C Lineage
Source: Insects. 2024 Jul 2;15(7):495. doi: 10.3390/insects15070495 (PMC11276638; doi:10.3390/insects15070495)
Supplement: Supplementary file 1 [file insects-15-00495-s001.zip › Table S2.pdf]

Table S2. Combination of absolute frequencies of *cox1* and *tRNA^{leu}-cox2* haplotypes

Haplotype	*tRNA*^{leu}*-cox2*													
cox1	C1a	C2c	C2d	C2d7	C2e	C2i	C2j	C2l	C2s	C2y	A1ha	A4na	A4s	Total
HpB01	1	0	8	1	48	1	1	1	3	0	0	0	0	64
HpB02	131	0	0	0	2	0	0	0	1	0	0	0	0	134
HpB03	22	41	0	0	0	0	0	1	0	1	0	0	0	65
HpB04	0	1	0	0	1	0	0	22	0	0	0	0	0	24
HpB05	0	0	3	0	1	0	0	0	0	0	0	0	0	4
HpB06	0	4	0	0	0	0	0	0	0	0	0	0	0	4
HpB07	0	0	0	0	4	0	0	0	0	0	0	0	0	4
HpB08	0	0	1	0	0	0	0	0	0	0	0	0	0	1
HpB09	0	0	0	0	1	0	0	0	0	0	0	0	0	1
HpB10	1	0	0	0	0	0	0	0	0	0	0	0	0	1
HpB11	0	1	0	0	0	0	0	0	0	0	0	0	0	1
HpB12	0	0	0	0	0	0	0	1	0	0	0	0	0	1
HpB13	0	0	0	0	1	0	0	0	0	0	0	0	0	1
HpB14	0	0	0	0	0	0	0	0	0	0	1	0	0	1
HpB15	0	0	0	0	0	0	0	0	0	0	0	0	1	1
HpB16	0	0	0	0	0	0	0	0	0	0	0	1	0	1
Total	155	47	12	1	58	1	1	25	4	1	1	1	1	308
